# One-step, Rapid and Green Synthesis of Multifunctional Gold Nanoparticles for Tumor-Targeted Imaging and Therapy

**DOI:** 10.1186/s11671-019-3232-3

**Published:** 2020-01-31

**Authors:** Hua Qin Yin, Guang Shao, Feng Gan, Gang Ye

**Affiliations:** 1 School of Chemistry, Sun Yat-Sen University, Guangzhou, 510275 People’s Republic of China; 2 Department of Gastroenterology, the First Affiliated Hospital of Jinan University, Guangzhou, 510630 People’s Republic of China

**Keywords:** One-step, Green, Multifunctional, Tumor imaging, Tumor therapy

## Abstract

Gold nanoparticles (GNPs) have always been used as doxorubicin (DOX) transport vectors for tumor diagnosis and therapy; however, the synthesis process of these vectors is to prepare GNPs via chemical reduction method firstly, followed by conjugation with DOX or specific peptides, so these meth•ods faced some common problems including multiple steps, high cost, time consuming, complicated preparation, and post-processing. Here, we present a one-step strategy to prepare the DOX-conjugated GNPs on the basis of DOX’s chemical constitution for the first time. Moreover, we prepare a multifunctional GNPs (DRN-GNPs) with a one-step method by the aid of the reductive functional groups possessed by DOX, RGD peptides, and nuclear localization peptides (NLS), which only needs 30 min. The results of scattering images and cell TEM studies indicated that the DRN-GNPs could target the Hela cells’ nucleus. The tumor inhibition rates of DRN-GNPs via tumor and tail vein injection of nude mice were 66.7% and 57.7%, respectively, which were significantly enhanced compared to control groups. One step synthesis of multifunctional GNPs not only saves time, materials, but also it is in line with the development direction of green chemistry, and it would lay the foundation for large-scale applications within the near future. Our results suggested that the fabrication strategy is efficient, and our prepared DRN-GNPs possess good colloidal stability in the physiological system; they are a potentially contrast agent and an efficient DOX transport vector for cervical cancer diagnosis and therapy.

## Introduction

Doxorubicin (DOX) is a commonly used anticancer drug which has been widely used in a variety of cancer chemotherapy, such as blood malignant tumor [1], a variety of adenocarcinoma [2–5], soft tissue sarcoma [6], and so on. However, long term and high dosages of DOX will lead to drug resistance [7], nausea [8], hair loss [9], and acute and chronic toxicity [10], which may lead to congestive heart failure [11]. Therefore, it is very necessary to develop a drug carrier with good biocompatibility and high drug loading ability. In recent years, a lot of nanomaterials, such as quantum dots [12, 13], chitosan [14, 15] silicon nanomaterials [16, 17], polymeric nanomaterials [18–20], fluorescent nanoparticles [21–24], and metal nanomaterials [25–30] were developed as DOX transport vectors for tumor diagnosis and therapy. Gold nanomaterials have been widely used due to their unique chemical and optical properties, low toxicity, good biocompatibility, and surface modification of control ability [31, 32] among these nanomaterials. So far, there are three kinds of approaches for the conjugation of DOX onto GNPs. The first is to conjugate DOX onto the surface of GNPs with the aid of hydrazine [25], 1-ethyl-3-[3-dimethylaminopropyl] carbodiimide hydrochloride (EDC) [26], pH-sensitive agent [27], DCC/NHS system [28]. The second is to incubate GNPs with DOX for at least 24 h [29]. The third is to replace citrate conjugated on the surface of gold nanoparticles (GNPs) by DOX [30]. Although these DOX-conjugated GNPs have been used in tumor therapy, the application is still limited due to the lack of sufficient specificity. Recently, El-sayed’s [27] group functionalized GNPs with DOX, RGD peptides, and nuclear localizing signal (NLS) peptides. The highlight of their work is that the GNPs enter tumor cells via receptor-mediated endocytosis of RGD peptides and then enter nucleus by the aid of NLS peptides, which makes DOX effectively interfere with DNA synthesis. Unfortunately, it needed at least 120 h by coupling peptides or drugs layer by layer on the GNPs, and each process needed at least 24 h. Similarly, all of the methods mentioned above faced some common problems such as multiple steps, high cost, complicated preparation, and post-processing. So it is necessary to develop a simple and rapid method for synthesis of multifunctional gold nanomaterials.

**Scheme 1:**
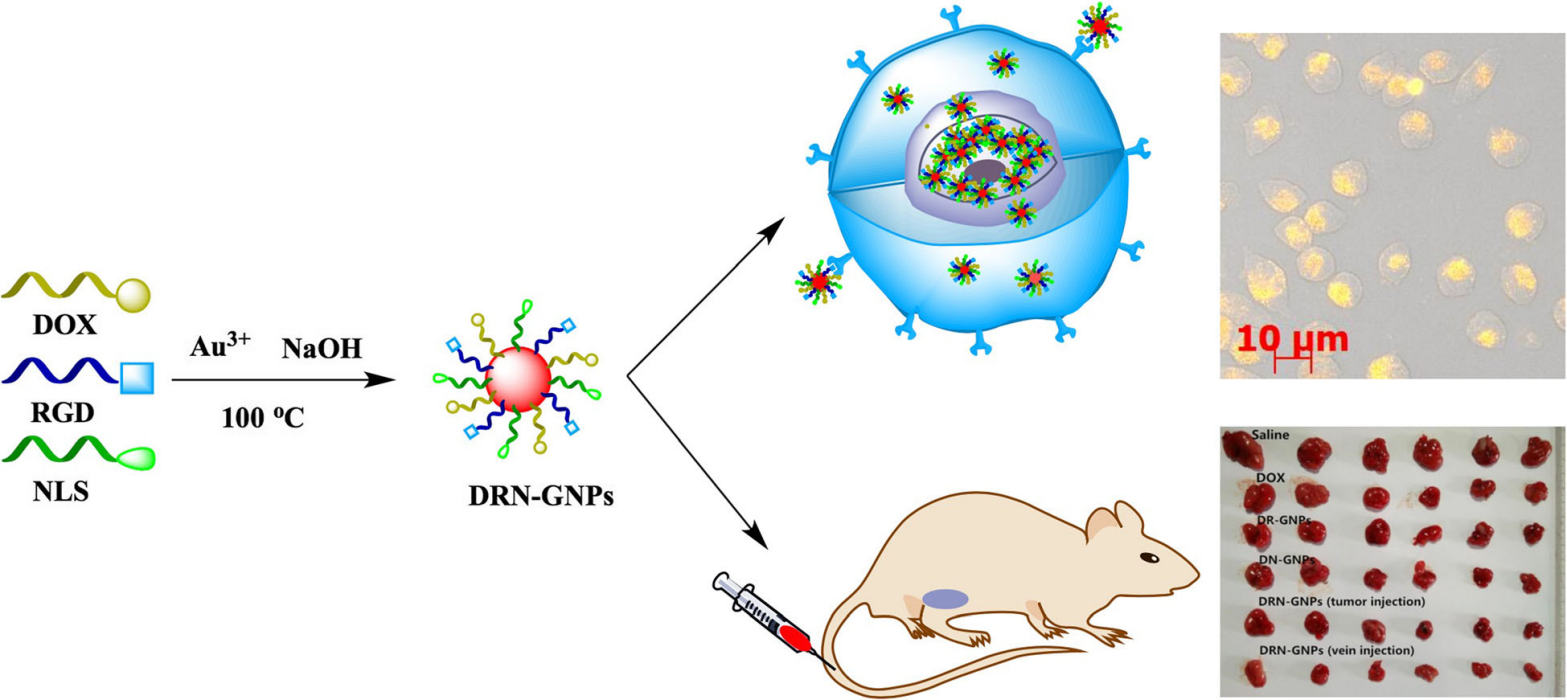
Schematic illustration of the synthesis of DRN-GNPs, the uptake by Hela cells and the tumor diagnosis of DRN-GNPs

In this paper, we presented a one-step strategy to prepare the DOX-conjugated GNPs on the basis of DOX’s chemical constitution for the first time. Moreover, we presented a convenient synthetic method to prepare a multifunctional DOX transport vector to make DOX work better, which can be used in tumor diagnosis and therapy. The key difference between our work from others is that we use the DOX, RGD peptides, and NLS peptides as reducing reagents and stabilizing reagents to fabricate the GNPs and make all of these three substances conjugate on the surface of the GNPs to form DRN-GNPs in the meantime. Our previous works [33–35] had proved that the RGD and other peptides can be used to reduce gold ions to fabricate gold nanomaterials. The N-terminal of the NLS can be modified by using cysteine cysteine tyrosine (CCY) sequence to construct CCYNLS, which can reduce gold ions while still keep NLS’s targeting effect [36]. We also found that DOX possesses two phenolic hydroxyl groups with strong reducibility under basic condition [37, 38], so it may reduce gold ions to form the GNPs too. Moreover, the anti-tumor effect of DOX might not be influenced because it embeds into DNA by the amino group of carbon 7 and the hydroxyl group of carbon 9 [39]. In the meantime, the sulfur, oxygen, and nitrogen on the peptides and DOX can be combined with the GNPs to keep the colloidal stable [33]. The results show that the DRN-GNPs were successfully fabricated and worked well in tumor imaging, diagnosis, and therapy (Scheme 1). To our knowledge, this is the first report for synthesis of DOX, RGD, and NLS peptides conjugated multifunctional GNPs with a one-step method, and their application in tumor diagnosis and therapy.

## Materials and Methods

### Materials

HAuCl_4_ 4H_2_O was purchased from Sinopharm Chemical Reagent Co., Ltd, cyclic RGD peptides (the sequence of amino acid is arginine-glycine-aspartate-tyrosine-glutamic acid (c(RGDyE))) and sodium hydroxide were the same as ref. [35]. Doxorubicin was purchased from Sangon Biotech (Shanghai) Co., Ltd. CCYNLS peptides (the sequence of amino acid is cysteine-cysteine-tyrosine-proline-proline-lysine-lysine-lysine-arginine-lysine-valine, CCYPPKKKRKV) were supplied by China Peptides Co., Ltd (Shanghai, China). The Hela cell line and the MCF-7 cell line were provided by Guangzhou Youdi Biological Technology Co. Ltd. Fetal bovine serum (FBS) and Dulbecco modified Eagle’s medium (DMEM) were purchased from GibcoBRL Life Technologies. The Proliferation and Cytotoxicity Assay Kit (MTT) was purchased from Beijing Dingguo Changsheng Biotechnology Co. Ltd.

### Synthesis of DOX-GNPs, NLS-GNPs, DR-GNPs, DN-GNPs, DRN-GNPs

For the preparation of the DOX-GNPs, DOX solution (0.2 mg/mL, 1.0 mL) was added into the chloroauric acid solution (0.86 mM, 1.0 mL) under magnetic stirring, then NaOH (2 M, 5 μL) was added to adjust pH. For the preparation of NLS-GNPs, CCYNLS peptides solution (0.5 mM, 1.0 mL) was added into the chloroauric acid solution (3.44 mM, 1.0 mL) under magnetic stirring, then NaOH (2 M, 20 μL) was added to adjust pH. For the preparation of DR-GNPs, DOX solution (0.2 mg/mL, 1.0 mL) and RGD peptides solution (1.0 mM, 1.0 mL) was added into the chloroauric acid solution (1.72 mM, 2.0 mL) under magnetic stirring, then NaOH (2 M, 15 μL) was added to adjust pH. For the preparation of DN-GNPs, DOX solution (0.2 mg/mL, 1.0 mL) and CCYNLS peptides solution (0.5 mM, 1.0 mL) was added into the chloroauric acid solution (1.72 mM, 2.0 mL) under magnetic stirring, then NaOH (2 M, 20 μL) was added to adjust pH. For the preparation of DRN-GNPs, solutions of DOX (0.2 mg/mL, 1.0 mL), CCYNLS peptides (0.5 mM, 1.0 mL), and RGD peptides (1.0 mM, 1.0 mL) was added into the chloroauric acid solution (1.72 mM, 3.0 mL) under magnetic stirring, then NaOH (2 M, 35 μL) was added to adjust pH. All of the reactions continued for 30 min at 100 °C in a reflux system and the pH value was 10–12. All the GNPs were purified by centrifugation at 55,000 rpm for 30 min (Optima MAX-TL, Beckman Coulter). After taking away the supernatant, the GNPs were redispersed in ultrapure water.

### Characterization

The characterizations of the GNPs’ spectra were implemented by using a UV 3150 Spectrophotometer (Shimadzu, Japan), a Nicolet Avatar FTIR model 330 spectrometer (Thermo, America), and a ESCALab250 X-ray photoelectron spectroscopy (XPS) with the same analytical conditions as ref. [35]. The transmission electron microscope (TEM) images of the GNPs were obtained from a TEM (JEM-2100F, JEOL, Japan) operated at an accelerating voltage of 200 kV.

### Dynamic Light Scattering and Zeta-Potential Measurements

A Zeta PALS Zeta Potential Analyzer (Brookhaven Instrument Corporation) was used to measure the dynamic light scatterings (DLS) of the DOX–GNPs, NLS-GNPs, DR-GNPs, DN-GNPs, and DRN-GNPs, which worked at 90° angle with a solid-state laser (λ = 670 nm) at room temperature. When equipped with an AQ-827 electrode, the analyzer was used to measure the GNPs’ zeta-potentials.

### ICP Analysis

The quantitative analysis of Au in the GNPs was the same as ref. [35].

### The Stability of DR-GNPs, DN-GNPs, and DRN-GNPs Dispersed in PBS, HCl, NaOH, NaCl Solution

We mixed 100 μL of the precipitated GNPs and 500 μL of high concentration of 0.2 M PBS, 0.5 M HCl, 0.5 M NaOH, and 1.0 M NaCl solution, respectively. Twelve hours later, we took their photos and tested their UV absorption spectra.

### Cell Culture

The Hela and MCF-7 cells were cultured in an incubator (humidified with 5% CO_2_ balanced air) at 37 °C for 24 h. The culture medium was DMEM with 10% FBS. The number of live cells are counted by a cell-count board.

### Cytotoxicity Assay of Free DOX and DRN-GNPs

The Hela and MCF-7 cells in the exponential phase were added to the wells of 96-well plates (about 5 × 10^3^ cells/well) and incubated for 24 h, respectively. Then the DRN-GNPs of concentrations 0, 20, 40, 60, 80, and 100 μg/mL (the corresponding DOX’s concentration was 0.0, 7.8, 15.6, 23.4, 31.2, and 39.0 μg/mL) were incubated with each well and incubated for 24 h at 37 °C, respectively. The control group was set by adding only PBS buffer solution, and its viability was set as 100%. The MTT (20 μL, 5 mg/mL) was added to each well and incubated at 37 °C for about 4 h. Then the culture medium was carefully suck out, and 150 μL of two dimethyl sulfoxide was added into per well for 10 min until the crystal was fully dissolved. A microplate reader (Thermo Multiskan Mk3) was used to measure each well’s OD value at 490 nm. Three wells for each group were prepared and the calculated values were expressed as mean ± S.D.

### Flow cytometry experiment

The Hela and MCF-7 cells in the exponential phase were added to each well of two 12-well plates (about 5 × 10^4^ cells/well) and incubated for 24 h, respectively. Then, the DRN-GNPs solution was added into each of the wells (final concentrations with 0, 20, 40, 60, 80, and 100 μg/mL) and incubated at 37 °C for 24 h, respectively. Removing the culture medium and adding Trypsin (containing EDTA) solution into the wells to digest the cells. Then removing the Trypsin solution from the wells, adding the culture medium in the wells, and transferring the cells into centrifuge tubes. After centrifugating at 1000 g for 5 min, the supernatant was removed and the cells in each tube were collected and counted in PBS solution. About 5–10 × 10^4^ of the cells were taken into another centrifugate tubes and were centrifugated at 1000 g for 5 min again. Removing the supernatant and adding 100 μL AnnexinV buffer solution into the tubes. Adding 5 μL of AnnexinV-FITC and 5 μL of propidium iodide into the tubes and mixing them with the cells gently. The tubes were incubated at room temperature (20–25 °C) for 15 min in the dark and then tested by a flow cytometer (BD Accuri C6).

### Optical Dark-Field Scattering Imaging and Transmission Electron Microscopy Studies on Cell Culture

The Hela and MCF-7 cells were implanted onto the 8-hole cell culture slide about 7 × 10^3^ cells for each well, respectively. The DRN-GNPs were added into each well with the final concentration of 35 μg/mL. After being incubated for 24 h, we removed physically and free absorbed GNPs by rinsing these cells with PBS solution for three times. Then, we fixed them with 4% paraformaldehyde for 20 min, coated with a few drops of glycerol, and sealed these 8-hole cell culture slides with another coverslips. A dark field microscope (Zeiss Imager Z1) was used to assess the cells in × 200 magnification and reflective mode. The exposure time for bright-field and dark-field images at a voltage of 5 V was 1 ms and 400 ms, respectively.

For TEM studies, Hela and MCF-7 cells were incubated with the DRN-GNPs (35 μg/mL) for 24 h in a cell culture flask, respectively. After incubating and sucking out the mixed solution of medium and DRN-GNPs, we rinsed the cells with PBS solution for three times and then digested them with trypsin. The cells were transferred in a centrifugal tube with a little volume of the DMEM medium. The cells were centrifugated (1500 rpm) for 10 min, the DMEM medium was sucked out carefully, and the 3% glutaraldehyde solution (usually 40 times the volume of materials) was added carefully into the tube to fix the cells in the bottom of the tube for more than 1 h. The cells were fixed further by adding 1% osmium tetroxide for 1 h, dehydrated with ethanol, and then embedded in Spurr (Sigma-Aldrich Co., USA). The cells were took out from the tube, cut into sections, stained with aqueous uranyl acetate and lead citrate, and then mounted on a copper grid (300 mesh). The specimens were measured using a FEI TECNAI-12 transmission electron microscope (working voltage 120 kV).

### Animal Experiments

#### Animal Subjects

Athymic female nude mice were purchased from Animal Experimental Center of Southern Medical University (Guangzhou, China) fo*r in vivo* tumor therapy test. All animal experiments were carried out in compliance with the Animal Management Rules of the Ministry of Health of the People’s Republic of China (Document NO. 55, 2001) and the guidelines for the Care and Use of Laboratory Animals of China Pharmaceutical University.

#### Therapeutic Efficacy of Free DOX and Its Conjugates in Tumor-Bearing Mice

Briefly, 36 nude mice (aged 5–6 weeks, weighed 16–18 g) were divided randomly into six groups. They were subcutaneously injected of Hela cells (5 × 10^6^ cells in PBS) into the right axillary fossa. Five days later, mice in the control group were injected by tail vein with saline solution (0.154 mol/L, 0.2 mL). Mice of free DOX, DR-GNPs, DN-GNPs, and DRN-GNPs groups were injected by tail vein too. In order to compare the DRN-GNPs’ anti-tumor efficacy between the intravenous injection and tumor injection, we set a group to be injected with equal amount of DRN-GNPs on the tumor site. These five groups maintained a dose of 0.3 mg/kg equivalent free or conjugated DOX in each mouse. Each mouse underwent tail vein or tumor injection once every day. The body weight and the tumor volume of each mouse was monitored every day over 14 days. To further investigate the therapeutic effects of DOX, DR-GNPs, DN-GNPs, and DRN-GNPs, the tumors of the six groups were excised for pathological analysis after 14 days of treatment. Tumors and the internal organs of heart, liver, spleen, lung, and kidney of the mice were isolated from the mice, fixed with 10% neutral-buffered formalin, and embedded in paraffin. The sliced tumor tissues were stained with Hematoxylin and Eosin (H&E) and examined by Olympus optical microscope.

## Results and Discussion

### Synthesis and Characterization of DOX-GNPs, NLS-GNPs, DN-GNPs, DR-GNPs, DRN-GNPs

In the synthesis of the DOX-GNPs, when the NaOH solution was added into the mixed solution of DOX and chloroauric acid solution, the color of the solution immediately changed from orange to shallow purple, it then became wine red at 100 °C in a reflux system under magnetic stirring in one minute, indicating the formation of the DOX-GNPs. The result indicated that the reduction ability of DOX is very strong because DOX possesses two phenolic hydroxyl groups (the No. 6 and No. 11 carbon) with strong reducibility under basic condition. The reaction continued for 30 min until the color do not change.

**Fig. 1:**
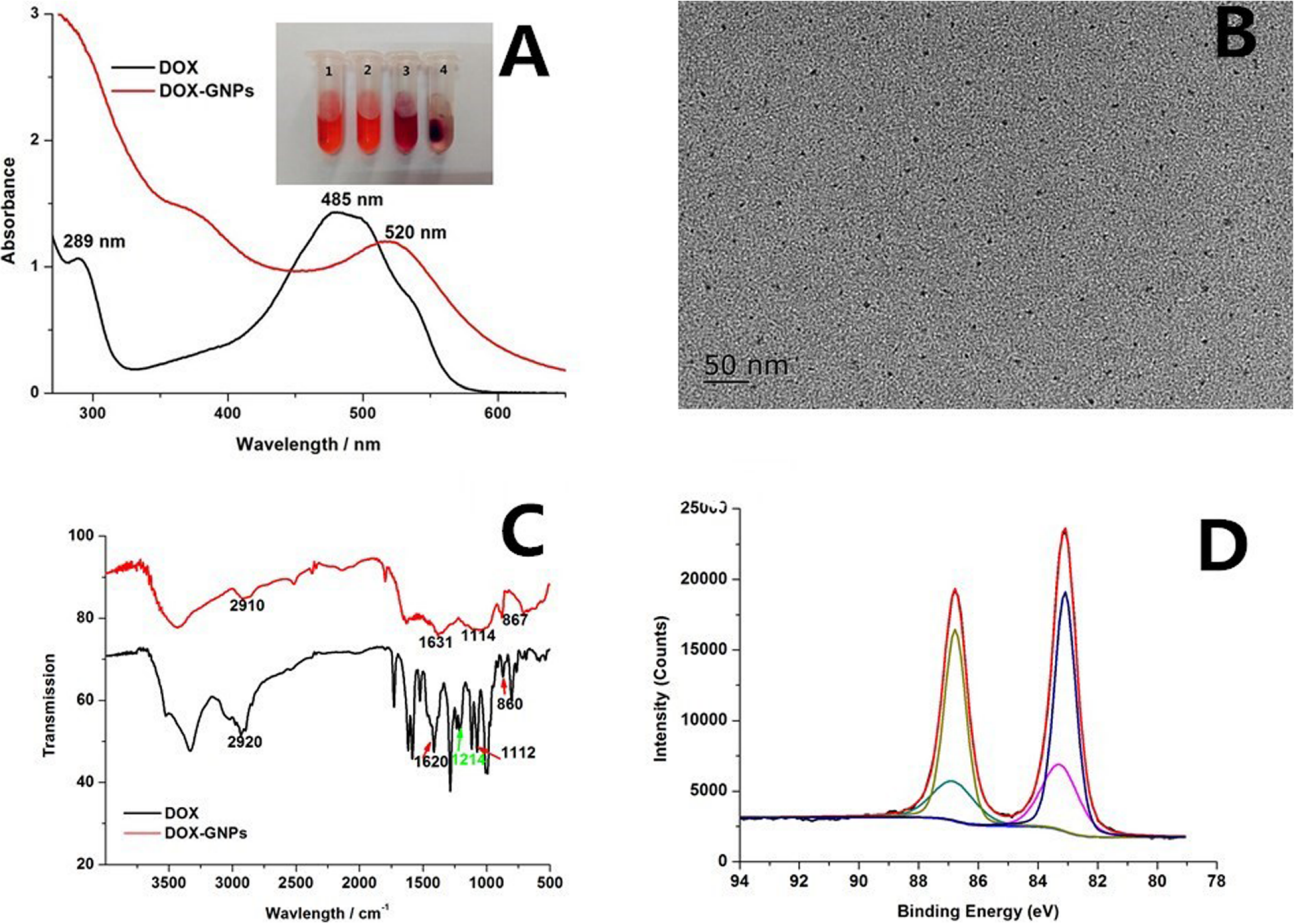
**a** UV-Visible absorption spectra of DOX and the as-prepared DOX–GNPs, the inset of (**a**) is the images of DOX (1, 2) and DOX-GNPs (3, 4) before (1, 3) and after centrifugation (2, 4). **b** TEM image of DOX–GNPs. **c** FTIR spectra of DOX and the as-prepared DOX–GNPs. **d** XPS spectrum of DOX–GNPs

In order to avoid confusing the DOX’s orange red color and the GNPs’ wine red color, we put forward more evidence by charactering their UV absorption spectra (as shown in Fig. 1a). DOX has a characteristic absorption peak near 485 nm and a peak at 530 nm; however, the peak at 480 nm disappeared in the spectrum of the product’s solution and a characteristic peak at 520 nm characteristic surface plasmon resonance (SPR) absorption peak of the GNPs arose. Even so, the formation of GNPs is still not completely determined between the 520 nm of the GNPs and 530 nm of the peak of DOX. Taking into account the GNPs will be precipitated with high-speed centrifugation, and DOX molecules will not, we can see purple precipitate in the product of the bottom of the centrifuge tube (inset of Fig. 1a(3-4)) and DOX do not have either (inset of Fig. 1a (1-2)).

DOX has red fluorescence under the irradiation of 365 nm ultraviolet light; however, the fluorescence of DOX disappeared after the synthesis of GNPs. There are two possible reasons; the first is that the GNPs’ extinction coefficient is very strong, they tend to quench the fluorescence of molecules when they are closely located to the surface of the GNPs (< 5 nm) [40]. If the distance increases to 20 nm or more, the nanoparticles’ plasmon field is too far to quench their fluorescence signal [41]. The other reason is that the fluorescent group of DOX was destroyed after playing the role of reduction agent. As shown in Fig. 1c, although the number of the DOX-GNPs’ infrared absorption peak is significantly less than that of the DOX, many of the characteristics of the DOX have been still retained, such as the characteristic IR absorption peaks at 2910 cm^−1^ (C-H, stretch vibration) [42], 1631 cm^−1^ (carbonyl stretch vibration) [43], 1114 cm^−1^ (C-O, C-N stretch vibration) [44], and 867 cm^−1^ (N-H, C-H, out-of-plane bending vibration) [45], respectively. Those representative peaks of DOX were also displayed in the spectra of the DOX-GNPs, indicated that the DOX was successfully bind to the DOX-GNPs. Interestingly, the peak at 1214 cm^−1^ characteristic peak for C-O stretch vibration of the phenolic hydroxyl group [46] disappeared in the spectra of DOX-GNPs. The phenolic group can reduce Au ions into GNPs, and then it was transported to carbonyl group. We speculate that the effect of DOX is not affected, because it embeds into DNA by the amino group of carbon 7 and the hydroxyl group of carbon 9 [39].

We can observe that the DOX-GNPs’ particle size is about 6 nm from the TEM results (Fig. 1b). The calculated ratio of Au (I) was 31.93% from the XPS spectrum of the DOX-GNPs (Fig. 1d), which is high enough to maintain the stability of the colloid. However, when the deposit of DOX-GNPs dispersed in 0.2 M PBS buffer solution with high-speed centrifugation after removing supernatant, the color of this solution become purple black, and there was visible insoluble floc. It indicated that the DOX-GNPs’ stability was not very well in the environment of the high salt concentration. It can be explained that DOX possesses only one amino group but does not possess sulfhydryl group, which is not enough for DOX to connect the surface of GNPs via Au-S and Au-N bond. So more efforts are needed if DOX-GNPs is used as contrast agent and anti-tumor material for tumor imaging and therapy.

**Fig. 2:**
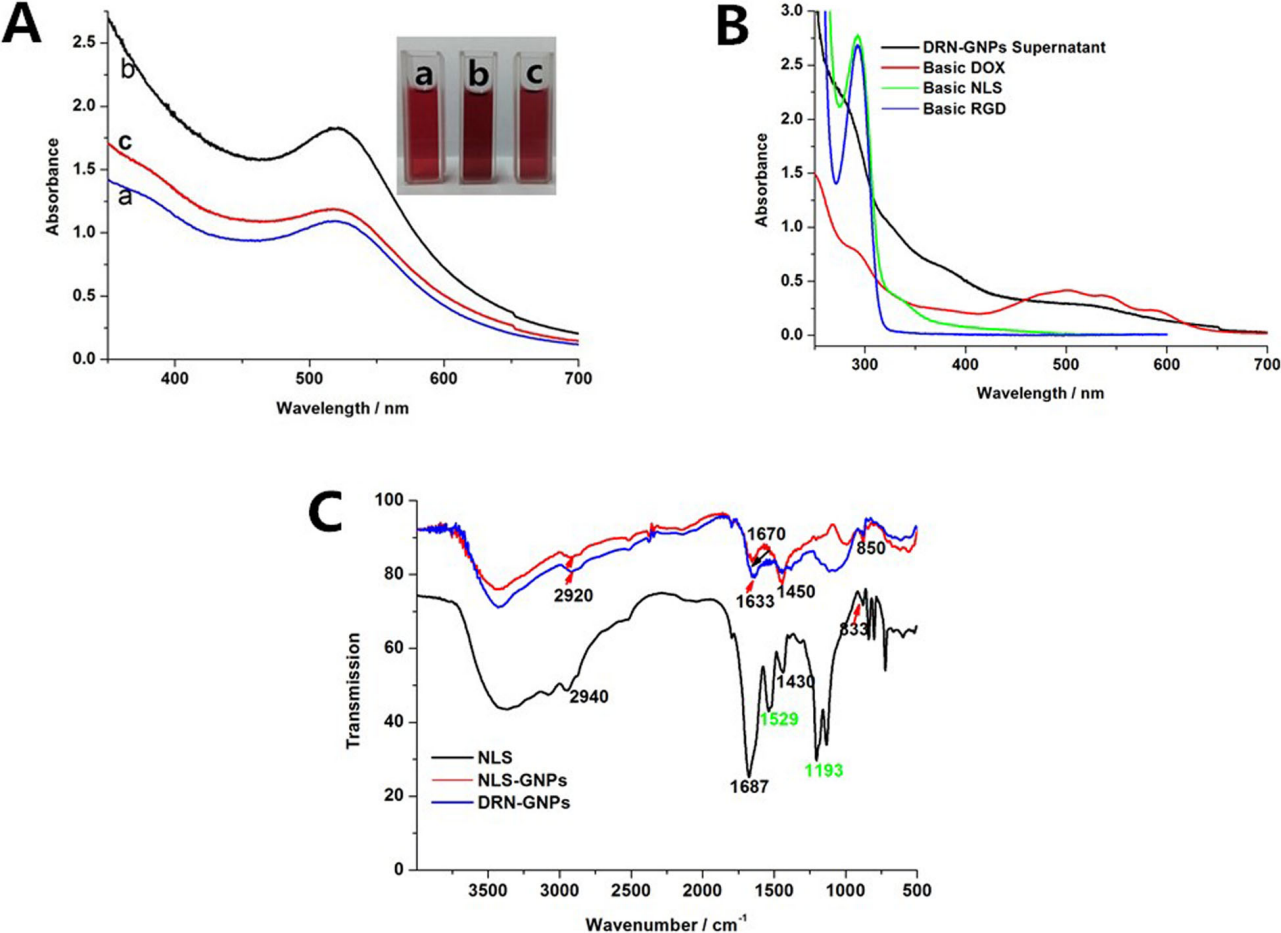
**a** UV-Visible absorption spectra and the photo images of DOX–GNPs (**a**), NLS-GNPs (**b**), and DRN-GNPs (**c**). **b** UV-Visible absorption spectra of basic DOX, RGD peptides, NLS peptides, and the supernatant of DRN-GNPs. **c** FTIR spectra of NLS peptides, NLS-GNPs, and DRN-GNPs. The distance between the GNPs’ spectrum and NLS peptides’ spectrum are enlarged for the contrast distinctly

We synthesized DRN-GNPs with DOX, the RGD peptides, and NLS peptides as reducing and stabilizing agents. The RGD peptides can specifically target some tumor cells overexpressed the α_v_β_3_ integrain, and the NLS peptides can specifically target the nucleus. Figure 2a shows the UV-Vis spectra of the fabricated DOX-GNPs, NLS-GNPs, DRN-GNPs; the characteristics of their surface plasmon absorption peak was 520–530 nm [33]. One can also see from the inset that the GNPs showed a bright red wine color. TEM images (Additional file 1: Figure S1) show that the particle size of NLS-GNPs and DRN-GNPs was about 10 nm and 5 nm, and the GNPs are distributed uniformly, no obvious coagulation was found.

In order to validate the synthesis of NLS-GNPs and DRN-GNPs, we characterized the FTIR spectra of NLS peptides and the GNPs in Fig. 2c. Some FTIR peaks of NLS peptides were found in the spectra of NLS-GNPs and DRN-GNPs, such as C-H stretching vibration absorption peak (2840–2972 cm^−1^) [47], C=O stretching vibration absorption peak (1630–1700 cm^−1^) [48], and =C-H in-plane bending vibration peak (1300–1475 cm^−1^) [49]; it indicated that the CCYNLS peptides were successfully conjugated on the surface of GNPs. We also found that the benzene skeleton vibration peak of tyrosine terminal in the NLS peptide at 1529 cm^−1^ and the phenolic hydroxyl C-O stretching vibration peak at 1193 cm^−1^ [49] disappeared in the infrared spectra of NLS-GNPs and DRN-GNPs, that means the benzene skeleton of tyrosine terminal was transported into a quinone structure after playing its role of reduction agent.

We centrifuge the DRN-GNPs with high speed, collected the supernatant fluid, which was almost colorless, and then tested whether there were left reactants via UV-Vis spectra in Fig. 2b. The UV absorption peaks of RGD and NLS peptides are derived from tyrosine residues, the absorption peak was located at 275 nm. However, the phenolic hydroxyl group became negative oxygen ions under alkaline conditions, which increases the electron density of benzene ring, the peak was red shifted to 292 nm. We did not see the characteristic peak in the UV-Vis spectrum of the DRN-GNPs’ supernatant, which means that both peptides took part in the reaction. DOX possesses three absorption peaks in the range of 450–600 nm, but the DRN-GNPs’ supernatant do not have those peaks. Besides that, no DOX was found in the DRN-GNPs’ supernatant because its color was light brown but the color of DOX under basic condition was transparent light purple. It can be concluded that the three materials almost react completely. Similarly, the DR-GNPs and DN-GNPs are also explained in this way, see Additional file 1: Figure S2.

**Fig. 3:**
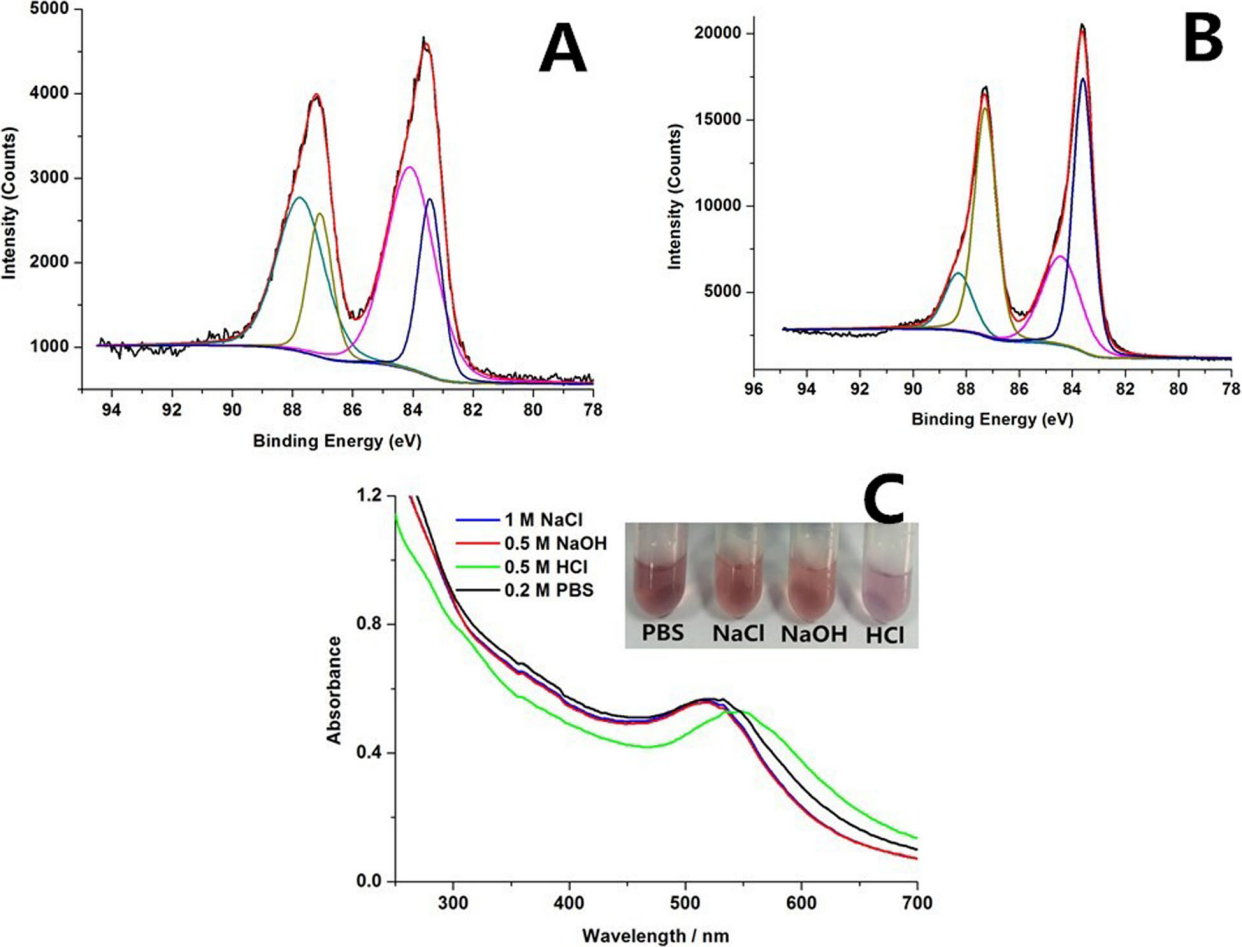
XPS spectrum of the Au 4f of the (**a**) NLS-GNPs, (**b**) DRN-GNPs. The Au 4f7/2 binding energy of the original spectra (black) and fitted results (red) could be deconvoluted into two components, Au(0)-blue curve and Au(I)-purple curve. UV-Visible absorption spectra and images of DRN-GNPs dispersed in PBS, NaCl, NaOH, and HCl, respectively (**c**).

We used XPS spectroscopy to examine the valence of Au for the NLS-GNPs and DRN-GNPs. As shown in Fig. 3a, b, two peaks with binding energies of around 83.6 eV and 87.0 eV are consistent with the emission of Au 4f7/2 and 4f 5/2 photoelectrons [33]. Their Au 4f7/2 binding energy position of both GNPs is nearly 84.0 eV. For the NLS-GNPs, it can be deconvoluted into Au (0) (83.5 eV) and Au (I) (84.1 eV), their peak area ratio is 57% and 43%, respectively. Similarly, for the DRN-GNPs, it can be deconvoluted into Au (0) (83.6 eV) and Au (I) (84.4 eV), the peak area ratios are 62% and 38%, respectively, thus they have a higher proportion of Au (I). The charge can form Au (I)-S complexes, which help to maintain the GNPs’ colloidal stability. Interestingly, we identified three types of S in the XPS spectra of NLS-GNPs, DN-GNPs, and DRN-GNPs in Additional file 1: Figure S3. The blue and black curves represent the oxidation states of sulfur, purple curves represent the S-H bond, and the green curve represents the Au-S bond. The formation of Au-S bond illustrated that the NLS peptides were successfully conjugated on the surface of these three GNPs.

Moreover, the peaks of N 1s, C 1s, and O 1s of XPS spectra demonstrated that the peptides and DOX were attached to GNPs (Additional file 1: Figure S4).

The XRD spectra (Additional file 1: Figure S5) shown that the crystal structure of these GNPs are face-centered-cubic crystalline structure, because all of their XRD spectra contained four peaks at 38.2°, 44.5°, 64.7°, and 77.8° corresponding to the (111), (200), (220), and (311) planes [33].

### Stability of DRN-GNPs

Dynamic light scattering (DLS) and zeta potential data of these five GNPs were displayed in Table 1; their hydrodynamic size is larger than that characterized by TEM, which can be attributed to a certain amount of hydrated molecules around the core of the water-soluble GNPs. Their zeta potentials were negative because of the carboxyl group conjugated on the surface of GNPs. The GNPs solution shows good colloidal stability when the absolute value of zeta potential is greater than 30 mV; the greater the absolute value, the better the stability. One can see that they all possess good colloidal stability from Table 1. In comparison, the NLS-GNPs’ value of zeta potential was the highest, that may be because their surface are full of NLS peptides which can form strong Au-S bond via the thiol group. The absolute value of DOX-GNPs’ zeta potential was close to 30 mV; it was consistent with the previously mentioned that the DOX-GNPs were not very stable when they were dispersed in PBS buffer solution. The spectra data were displayed in Additional file 1: Figure S6.

**Table 1:** The zeta-potential, hydrodynamic size, and polydispersity index of DOX-GNPs, NLS-GNPs, DN-GNPs, DR-GNPs, and DRN-GNPs. Data are provided with mean ± S.D. (*n* = 5)

	Zeta potential	Hydrodynamic size	Polydispersity index
DOX-GNPs	− 31.5 ± 6.2	46.1 ± 1.9	0.341 ± 0.031
NLS-GNPs	− 41.8 ± 2.9	88.8 ± 4.1	0.341 ± 0.018
DN-GNPs	− 33.6 ± 2.5	77.4 ± 3.2	0.352 ± 0.010
DR-GNPs	− 36.7 ± 5.8	17.2 ± 0.4	0.232 ± 0.013
DRN-GNPs	− 38.4±3.2	61.0 ± 5.3	0.339 ± 0.007

The changes of the GNPs’ characteristic surface plasmon resonance (SPR) bands can be used to determine the aggregation of GNPs by utilizing UV-Vis spectrometry [49]. We measured their UV-Vis spectra and take the photographs of the DRN-GNPs (Fig. 3c) when they were dispersed in high salt (1 M NaCl and 0.2 M PBS), strong acid (0.5 M HCl), and strong base (0.5 M NaOH) solutions. Neither solution color change nor UV-vis spectra shift was observed under various harsh conditions except in the HCl solution, in which their color changed to slight purple and the ultraviolet absorption peak red shift about 20 nm, which means GNPs coagulated slightly under strong acid condition. The reason might be that the negatively charged GNPs colloidal stability was affected under strong acidic condition, but it was not influenced in the neutral PBS buffer and alkali environment. We can conclude that the DRN-GNPs are highly stable under high salt and alkali conditions, indicating that our synthesized DRN-GNPs were expected to be used for in vitro cell imaging and in vivo anti-tumor therapy.

### Plasmonic Dark-Field Scattering Imaging of Tumor Cells and Cell TEM Microscopy

**Fig. 4:**
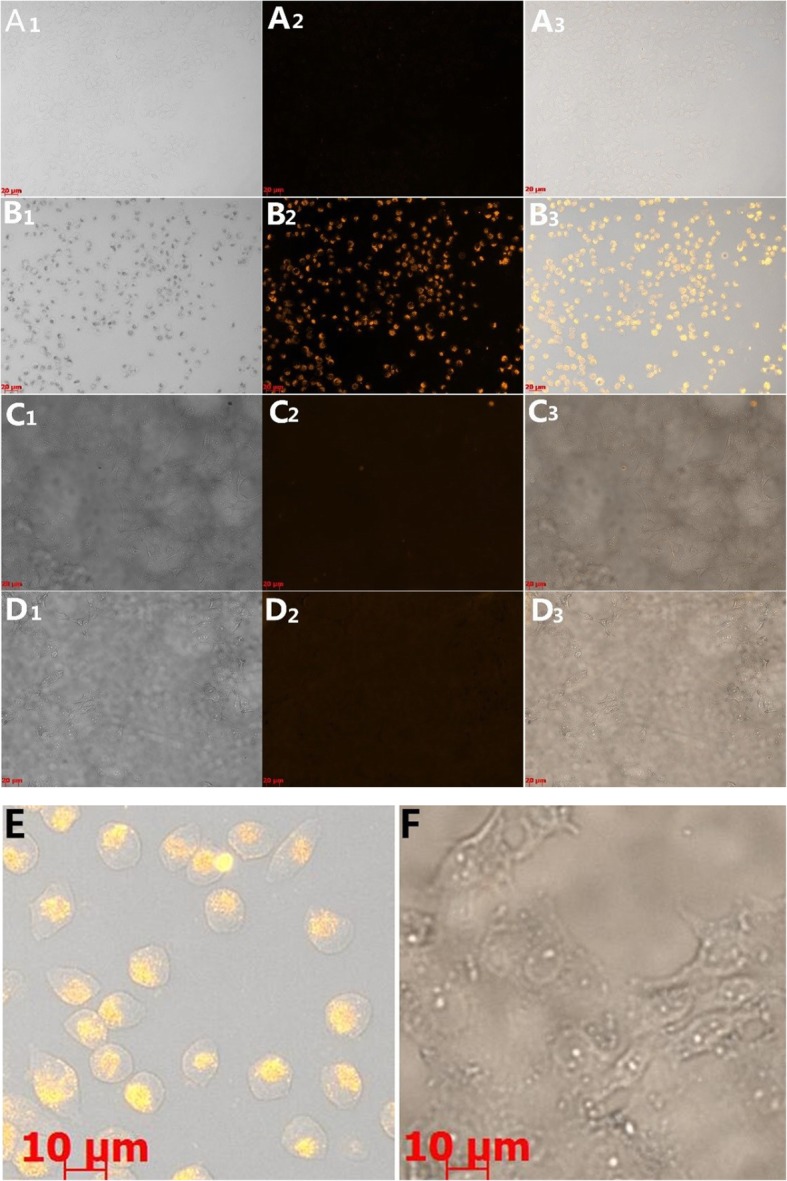
The contents of the rows are listed as follows: “PBS buffer” represents the cells incubate with PBS buffer, “DRN-GNPs” represents the cells incubate with DRN-GNPs. The subscript of “1” represents the bright field images of cells, “2” represents the dark field images of cells, and “3” represents the overlapping images (bright-field and dark-field) of cells. All of the scale bars are 20 μm. The picture of E is a small region chosen from B_3_ in Fig. 4, the picture of F is a small region chosen from D_3_ in Fig. 4, the scale bars are 10 μm

**Fig. 5:**
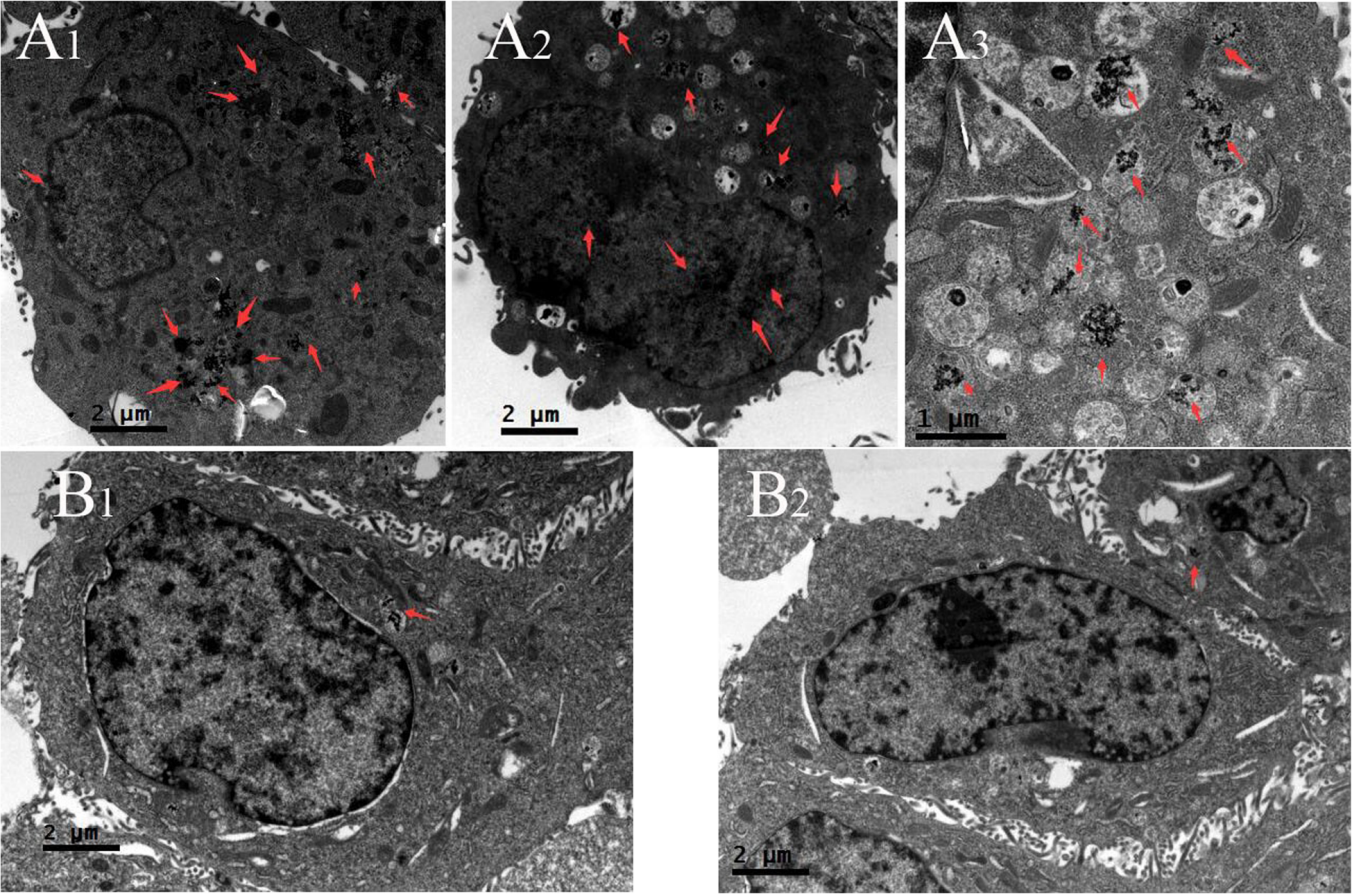
TEM images of DRN-GNPs incubated in Hela and MCF-7 cells for 24 h. A_1_, A_2_, and A_3_ are corresponding to HeLa cells while B_1_ and B_2_ are to MCF-7 cells

For the specific targeted imaging of tumor cells with DRN-GNPs, we selected the Hela cells as test group and the MCF-7 cells as the control group. The reason is that the Hela cells overexpress the integrin α_v_β_3_ [50] but the MCF-7 cells do not, so it has no specific binding of RGD; it is a good control group [51]. The uptake of DRN-GNPs by these two cell lines after incubation for 24 h with a final concentration of 35 μg/mL was assessed by an upright fluorescence microscopy in the dark-field model. The cells being treated without GNPs do not show plasmonic light scattering (Fig. 4 A-Hela cells, 5C-MCF-7 cells). Images of Hela cells being treated with the DRN-GNPs show that scattering signals from the GNPs is heavily localized at the nucleus (Figs. 4b and 5e). However, we could hardly see the scattering light from the MCF-7 cells after they were incubated with the DRN-GNPs, even though little amount of GNPs might have entered the tumor cells through the tumor cells’ passive enhanced permeation and retention effect (EPR effect) (Figs. 4d and 5f), the enhanced permeability and retention (EPR) effect is a unique phenomenon of solid tumors related to their anatomical and pathophysiological differences from normal tissues. For example, angiogenesis leads to high vascular density in solid tumors, large gaps exist between endothelial cells in tumor blood vessels, and tumor tissues show selective extravasation and retention of macromolecular drugs [52]. So they might not be clearly detected under the same experimental condition. The results demonstrated that the endocytosis of Hela cells via receptor-mediated endocytosis was facilitated by the RGD peptides on the GNPs’ surface. The NLS peptides could also maintain their activity to specifically targeted nucleus. So our synthesized DRN-GNPs could be a very potential contrast agent for tumor targeted imaging.

Table 2 shows the ratio values between the cells’ densitometric mean grey and the background in the same image, which has a positive correlation with the taken up amounts of DRN-GNPs by these two cell lines. The ratios of the cells incubated with PBS buffer and the MCF-7 cells were nearly 1.0, the brightness of cells was nearly close to the background. The ratio of Hela cells incubated with DRN-GNPs was 4.80, that means the synthesized DRN-GNPs could be specifically targeted and entered the tumor cells overexpressed the integrin α_v_β_3_.

**Table 2:** Comparison of DRN-GNPs taken up by Hela and MCF-7 cells. The ratio of the value of the cells’ densitometric mean (grey) to that of the background in the same image

	Ratio of mean grey (cell / background)
Hela (PBS)	1.05
Hela (DRN-GNPs)	4.80
MCF-7 (PBS)	1.12
MCF-7 (DRN-GNPs)	1.01

To confirm receptor-specific internalization of the DRN-GNPs, we investigated the cellular uptake of the DRN-GNPs by Hela and MCF-7 cells utilizing the TEM technique (see Fig. 5). There was a large amount of DRN-GNPs localized in the nucleus and cytoplasm regions of α_v_β_3_-positive Hela cells (Fig. 5(A_1_- A_3_)). On the other hand, only a negligible amount of particles was found inside the cytoplasm regions of α_v_β_3_-negative MCF-7 cells (Fig. 5(B_1_-B_2_)), they might be accumulated in the MCF-7 cells by means of the passive targeting EPR effect. We found that there were high contrast black continuous regions in the MCF-7 cells’ nucleus; they were uranyl acetate, which stained the nucleus, and they were different from the granular particles in the nucleus of Hela cells. The TEM images are consistent with the dark-field imaging results, indicating that the RGD and NLS peptides on the surface of GNPs facilitated the DRN-GNPs targeting Hela cells’ nucleus. So our synthesized DRN-GNPs could be used as a perfect contrast agent for tumor targeted imaging and diagnosis.

### Cellular Therapeutic Efficacy Evaluation

**Fig. 6:**
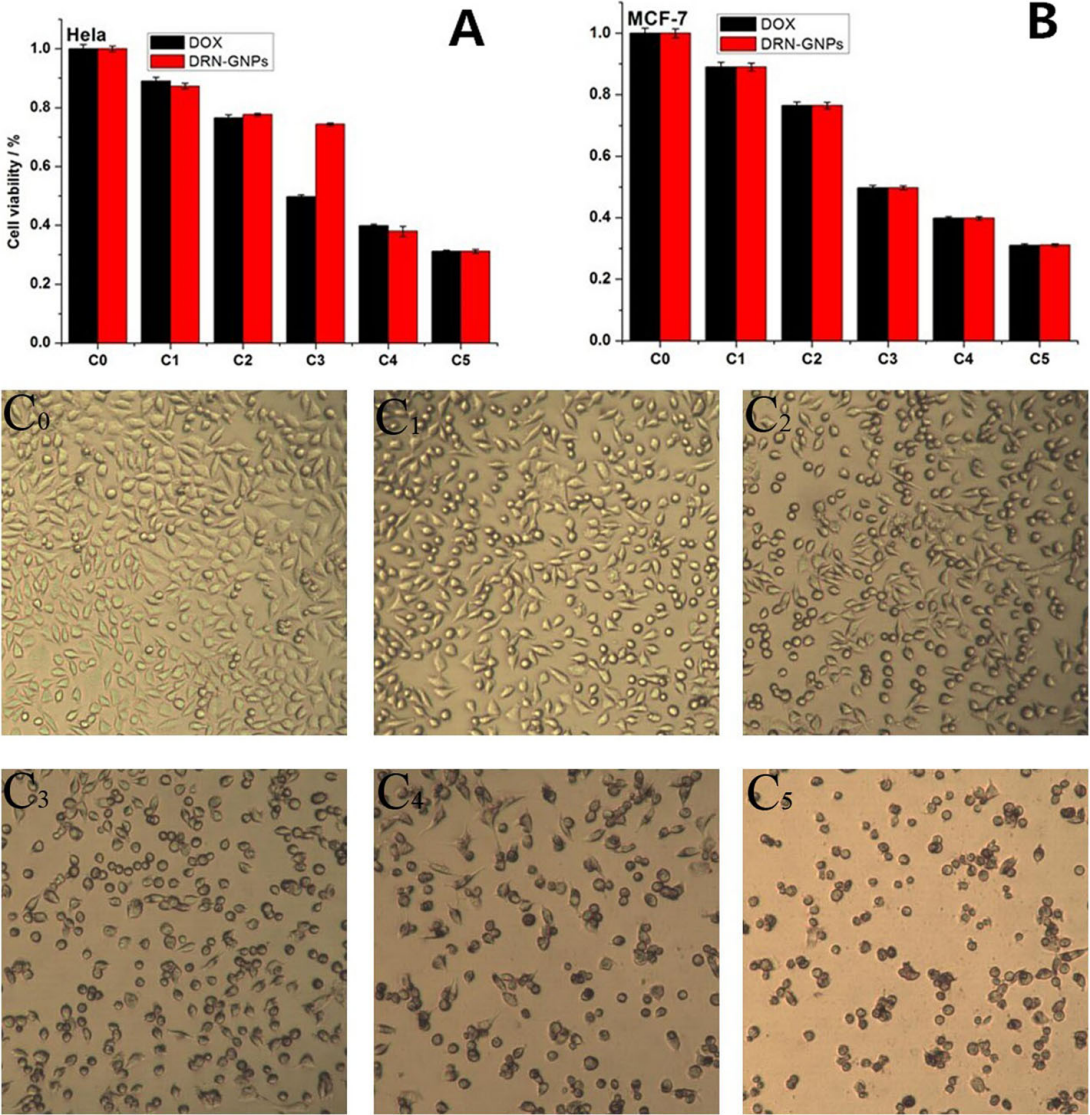
MTT assay was used to qualitatively display in vitro anti-tumor activity of DOX and DRN-GNPs on Hela (**a**) and MCF-7 cells (**b**) for 24 h (100%). The concentrations of C_0_-C_5_ are 0, 20, 40, 60, 80, 100 μg/mL for DRN-GNPs; the corresponding DOX’s concentrations are 0, 7.8, 15.6, 23.4, 31.2, 39.0 μg/mL. Data are represented as means ± standard deviations of triplicate samples (*n* = 3). Images of Hela cells incubated with different concentration of DRN-GNPs (**c**)

To evaluate the therapeutic efficacy of DOX and DRN-GNPs at cell level, MTT assay was firstly carried out to study cell viability of Hela and MCF-7 cells under the treatment of these two samples for 24 h. DRN-GNPs demonstrated almost the same inhibition effect by killing nearly 70% of cells at the same DOX’s concentration (39.0 μg/mL, Fig. 6(A, B)). In line with the MTT results, when the concentration of DRN-GNPs was 40 μg/mL, the number and state of the Hela and MCF-7 cells were relatively good, so the concentration of 35 μg/mL was the suitable concentration for imaging. We observed that when the DRN-GNPs’ concentration was high to 60 μg/mL (Fig. 6(C_3_)), the Hela cells became necrosis obviously. When the concentration of DRN-GNPs goes high to 100 μg/mL, salient reduction of the number cells can be observed and clear cellular morphological change as the characteristic of necrosis was observed. The same concentration of conjugated DOX still exhibited nearly the same anti-tumor efficacy compared with the free DOX. These results indicated that our synthesized DRN-GNPs could be used not only as a contrast agent but also as a good drug delivery system.

**Fig. 7:**
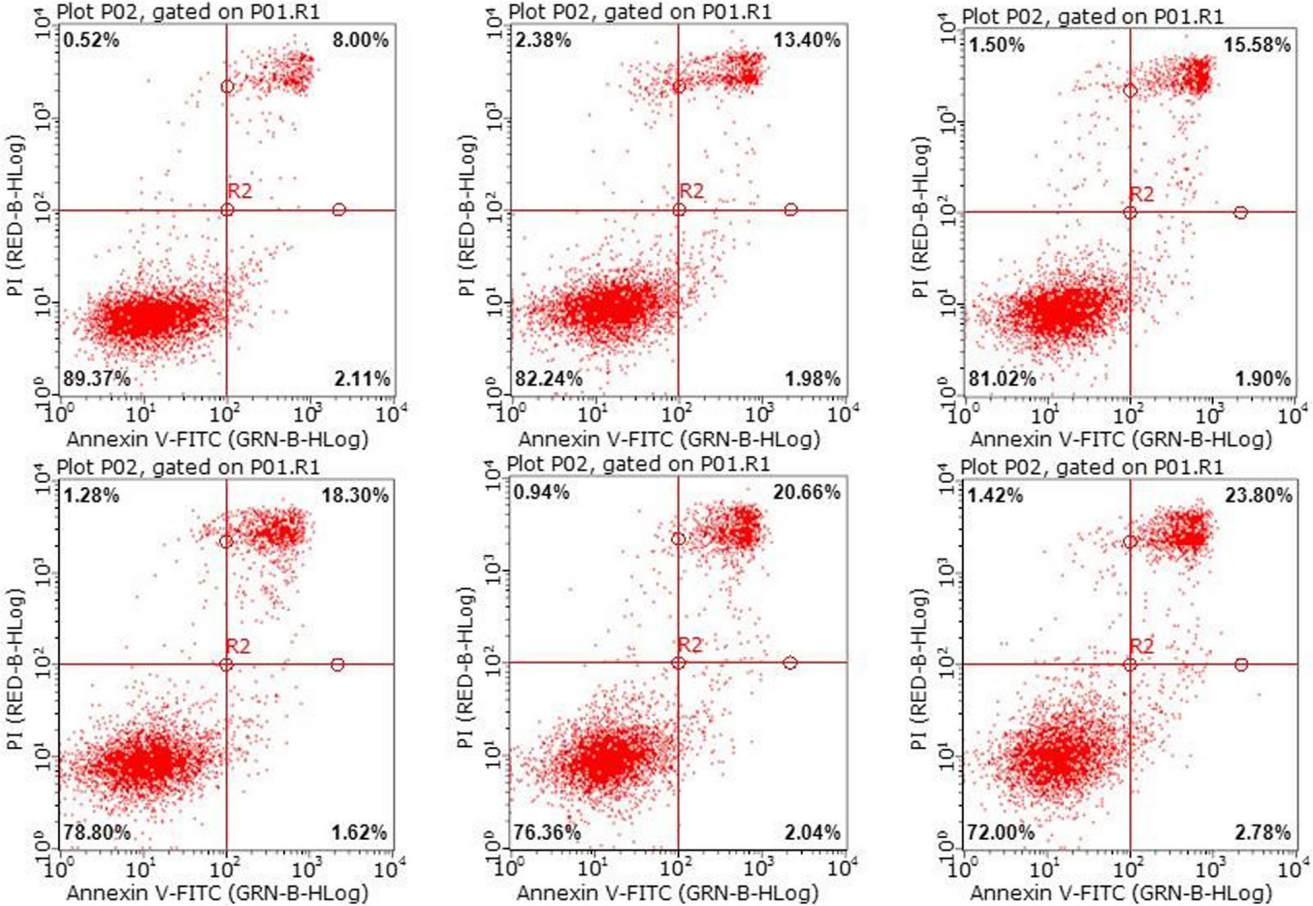
Representative two-dimensional contour density plots to determine fractions of live, apoptotic, and necrotic cells, when exposed to DRN-GNPs at different concentrations (0–100 μg/mL) for 24 h, respectively. Cell necrosis and apoptosis were measured by using PI and Annexinv-FITC dyes

The result detected by the flow cytometry in Fig. 7 also demonstrates the anti-tumor efficacy of DRN-GNPs. Viability of Hela cells when exposed to DRN-GNPs of different concentrations was measured by flow cytometry based assays. Two modes of cell death, apoptosis and necrosis, were measured using Annexinv-FITC and propidium iodide (PI) dyes, respectively (Fig. 7). Similar to the results of MTT assay, the data showed that DRN-GNPs induced cell necrosis was dose-dependent. The proportion of necrotic cells was 8% without incubating with DRN-GNPs; when the concentration was high to 100 μg/mL, the proportion of necrotic cells was 23.84%; that means our synthesized DRN-GNPs could kill Hela cells efficiently. The difference between the results of MTT and flow cytometry can be explained that the difference of cell number and the methods.

### Therapy Evaluation of DRN-GNPs in Tumor-Bearing Mice

**Fig. 8:**
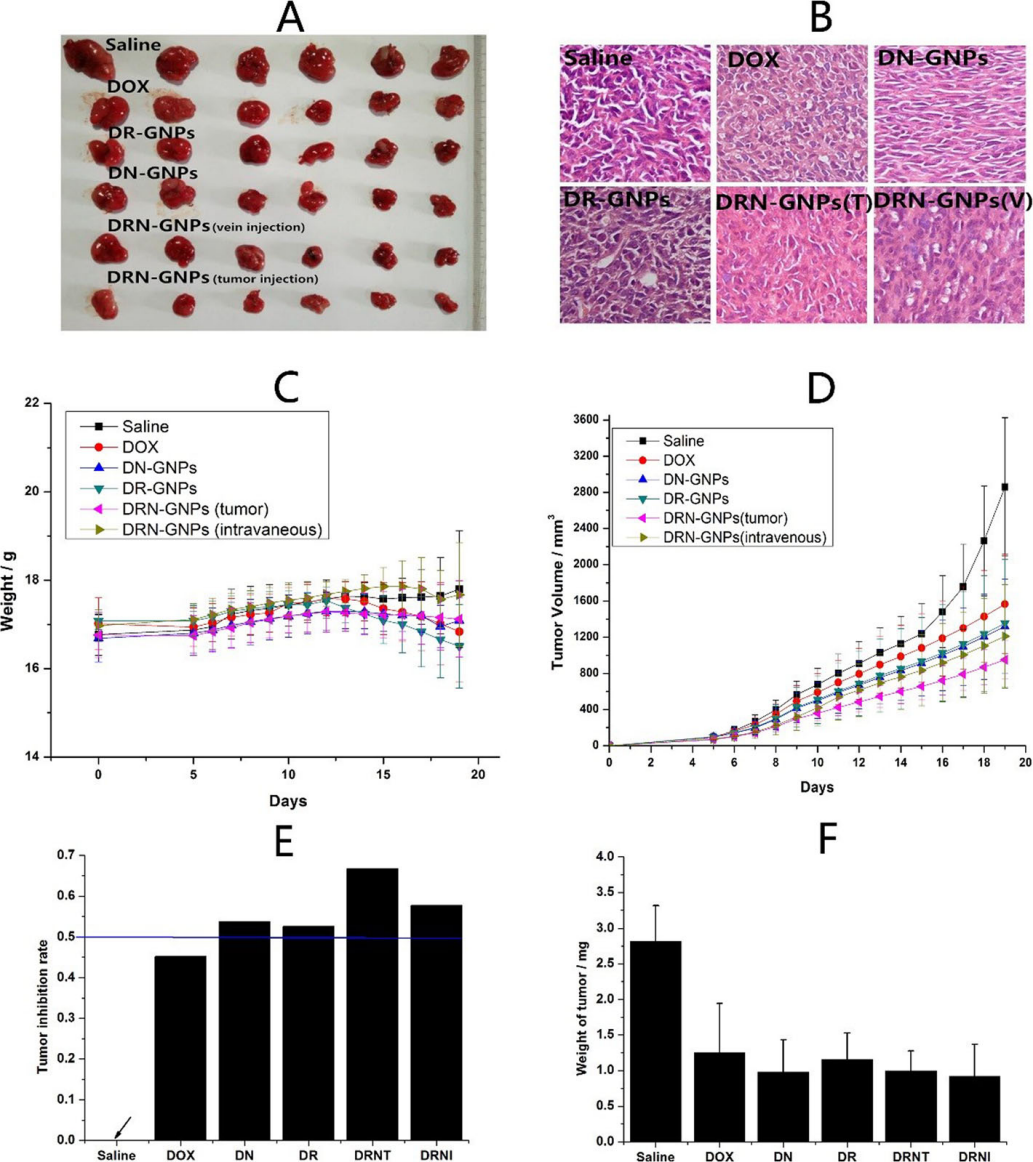
**a** Tumor images isolated from tumor-bearing mice after treatment of saline, free DOX, DN-GNPs, DR-GNPs, and DRN-GNPs (tumor injection and intravenous injection). **b** The histological images of tumors of the mice after treated with saline, free DOX, DN-GNPs, DR-GNPs, and DRN-GNPs (tumor injection and intravenous injection). **c** Body weight, tumor volume (**d**), tumor inhabitation rate (**e**), and tumor’s weight (**f**) of tumor-bearing mice during 14 days treatment; data are represented as means ± standard deviations (*n* = 6)

**Fig. 9:**
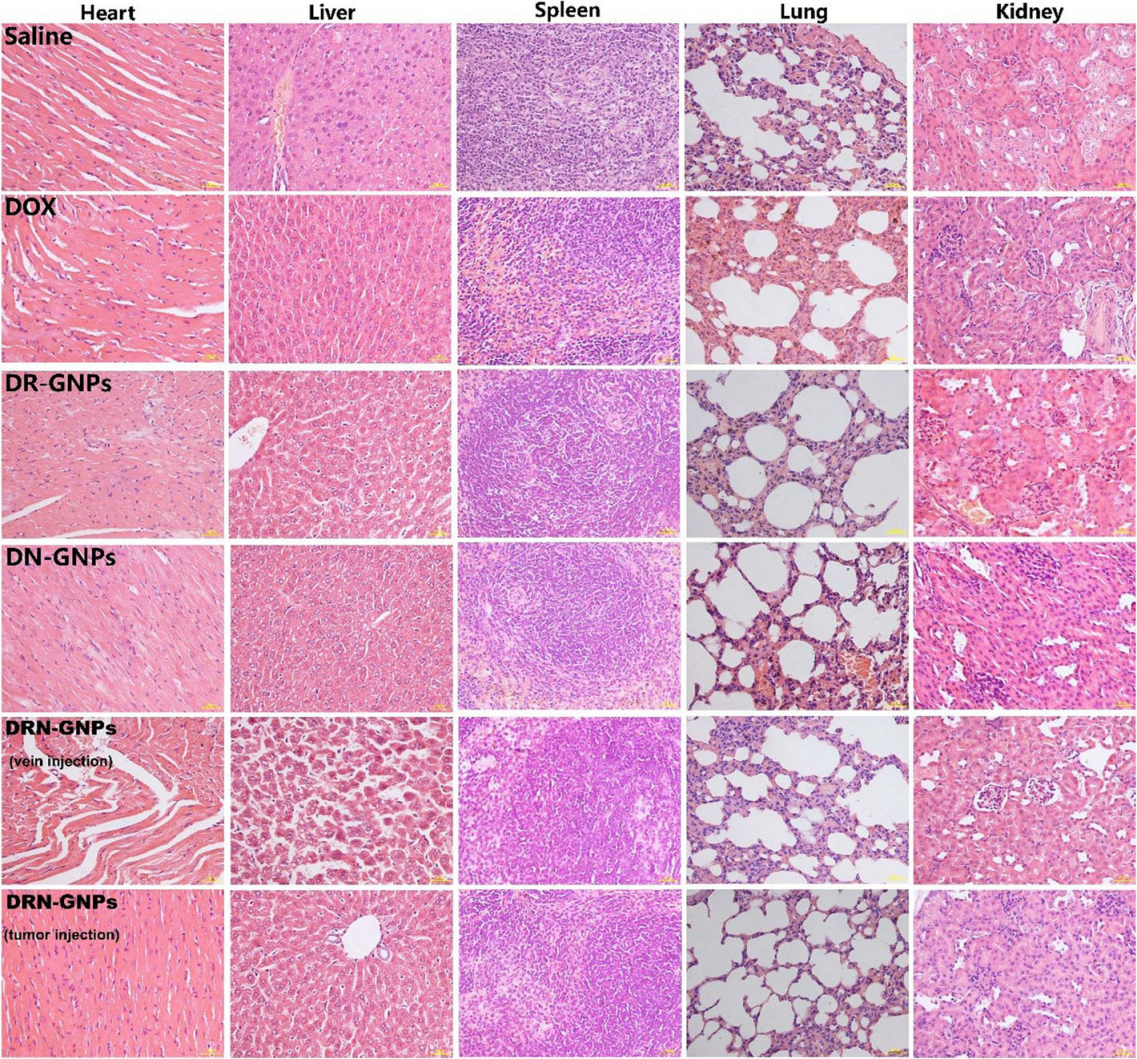
The histological images of the main organs (heart, liver, spleen, lung, and kidney) of the mice after treated with saline, free DOX, DR-GNPs, DN-GNPs, and DRN-GNPs (tumor injection and intravenous injection)

To further determine whether DRN-GNPs induced a combined therapeutic effect on tumor cells in vivo, we evaluated the anti-tumor effect of DRN-GNPs in tumor-bearing mice for long-term intravenously injection of drugs. The saline group and the drugs of free DOX, DR-GNPs, and DN-GNPs intravenously injected groups were set as the control groups, in which the concentration of conjugated DOX was the same as that of the free DOX. We observed that the tumor sizes of DRN-GNPs-treated groups were obviously smaller than that of the saline, DOX, DN-GNPs, and DR-GNPs-treated groups (Fig. 8a). The tumor volume and body weight of the mice were monitored every 2 days. Significant variation of tumor volume and tumor weight among all of the treated groups is shown in Fig. 8d, f; the tumor volumes and tumor weight of DN-GNPs and DR-GNPs-treated groups were smaller than that of the free DOX-treated group, but still larger than that of the DRN-GNPs-treated groups because of the less targeted activity. That might be because DOX molecules are small, lack targeting, and they have a short half-life in vivo; therefore, they might be cleared out quickly. However, DRN-GNPs possess of strong stability in the blood system, long half-life, and highly targeted ability, so they can be targeted to the tumor site, and then play the anti-tumor effect of DOX. Between the DRN-GNPs intravenously injected and tumor-injected groups, the anti-tumor efficacy of the latter group is better than that of the former group because the drugs do not need a complex system of blood circulation into the focus. The tumor inhibition rate of DOX is less than 50%, and the rates of all of the DOX-conjugated GNPs are larger than 50%; they can be high to 66.7% and 57.7%, respectively. The HE stain results showed that there was a certain degree of damage for liver organs in the intravenous injected DRN-GNPs group (Fig. 9). Furthermore, the bio-toxicity of DOX, DR-GNPs, and DN-GNPs on tumor-bearing mice were also assessed by histological analysis. As shown in Fig. 8b, tumor cell volume of saline group is comparatively larger than that of treatment groups, and tumor cells demonstrate with more mitotic figures. All the results showed that the tumor inhibition effect of DRN-GNPs was the best among the GNPs. Thus, our synthesized DRN-GNPs could be used as a perfect DOX delivery system for tumor therapy.

## Conclusion

In this study, we have successfully developed a one-step method to prepare the multifunctional DRN-GNPs in about half an hour. The GNPs have also been successfully applied to the tumor targeted imaging and tumor therapy both in vitro and in vivo*.* The success shows that it is possible to make a fully use of the reducing and stabilizing ability of the DOX, RGD peptides, and CCYNLS peptides, which will make the GNPs’ synthesis process simple and efficient. More importantly, the one-step synthesized DRN-GNPs still possess good colloidal stability in the physiological system, and the peptides conjugated on the surface remained the targeted ability and DOX still possesses its anti-tumor ability. To our knowledge, this is the first report for synthesis of DOX, RGD and CCYNLS peptides conjugated multifunctional GNPs with a one-step method, and their application in tumor imaging, diagnosis, and therapy. This strategy is in line with the development direction of green chemistry, and it would lay the foundation for large-scale applications within the near future. We expect that our report will provide a new way for the one-step synthesis of multifunctional nanomaterials used for tumor imaging and therapy.

## Supplementary information

**Additional file 1: Figure S1.** TEM images of the NLS-GNPs, DRN-GNPs, DR-GNPs and DN-GNPs. **Figure S2.** UV-Visible absorption spectra of basic DOX, RGD peptides, NLS peptides and the supernatant of DN-GNPs and DR-GNPs. **Figure S3.** XPS spectra of the S from NLS–GNPs, DN-GNPs and DRN-GNPs. **Figure S4.** XPS spectra of the C, N, O from DOX-GNPs, NLS–GNPs, DR-GNPs, DN-GNPs, and DRN-GNPs. **Figure S5.** XRD figures of DOX-GNPs, NLS-GNPs, DR-GNPs, DN-GNPs and DRN-GNPs. **Figure S6.** The dynamic light scattering and zeta potential figures of the GNPs. **Figure S7.** TEM images of DRN-GNPs incubated in Hela and MCF-7 cells for 24 h.

## Data Availability

All datasets are presented in the main paper or in the additional supporting files.

## Abbreviations

CCYNLS peptides: The sequence of amino acid is cysteine-cysteine-tyrosine-proline-proline-lysine-lysine-lysine-arginine-lysine-valine
Cyclic RGD peptides: The sequence of amino acid is arginine-glycine-aspartate-tyrosine-glutamic acid
DOX: Doxorubicin
DR-GNPs: DOX-RGD-GNPs, DN-GNPs: DOX-NLS-GNPs, DRN-GNPs: DOX-RGD-NLS GNPs
GNPs: Gold nanoparticles

## References

[1] Lipshultz SE, Colan SD, Gelber RD, Perezatayde AR (1991). Late cardiac effects of doxorubicin therapy for acute lymphoblastic leukemia in childhood. N Engl J Med.

[2] Cullinan SA, Moertel CG, Fleming TR, Rubin JR (1985). A comparison of three chemotherapeutic regimens in the treatment of advanced pancreatic and gastric carcinoma. Fluorouracil vs fluorouracil and doxorubicin vs fluorouracil, doxorubicin, and mitomycin. JAMA.

[3] Qi N, Tang B, Liu G, Liang XS (2017). Poly(γ-glutamic acid)-coated lipoplexes loaded with Doxorubicin for enhancing the antitumor activity against liver tumors. Nanoscale Res lett.

[4] Song YF, Liu DZ, Cheng Y, Liu M (2015). Dual subcellular compartment delivery of doxorubicin to overcome drug resistant and enhance antitumor activity. Sci Rep.

[5] ZolataH AH, Abbasi-Davani F (2014). Radio-immunoconjugated, Dox-loaded, surface-modified superparamagnetic iron oxide nanoparticles (SPIONs) as a bioprobe for breast cancer tumor theranostics. J Radioanal Nucl Ch..

[6] Santoro A, Tursz T, Mouridsen H, Verweij J, Steward W (1995). Doxorubicin versus CYVADIC versus Doxorubicin plus ifosfamide in first-line treatment of advanced soft tissue sarcomas: a randomized study of the European organization for research and treatment of cancer soft tissue and bone sarcoma group. J Clin Oncol.

[7] Pastan I, Gottesman M (1987). Multiple-drug resistance in human cancer. N Engl J Med.

[8] Kim NK, Park YS, Heo DS, Suh C, Kim SY, Park KC (1993). A Phase III randomized study of 5-fluorouracil and cisplatin versus 5-fluorouracil, doxorubicin, and mitomycin c versus 5-fluorouracil alone in the treatment of advanced gastric cancer. Cancer.

[9] Lammer J, Malagari K, Vogl T, Pilleul F, Denys A, Watkinson A (2010). M. Pitton, Prospective randomized study of doxorubicin-eluting-bead embolization in the treatment of hepatocellular carcinoma: results of the PRECISION V Study. Cardiovasc. Intervent Radiol.

[10] Buzdar AU, Marcus C, Blumenschein GR, Smith TL (1985). Early and delayed clinical cardiotoxicity of doxorubicin. Cancer.

[11] Von Hoff DD, Layard MW, Basa P, Davis HL (1979). Risk factors for doxorubicin-lnduced congestive heart failure. Ann Intern Med.

[12] Lin B, Yu YH (2015). Phosphorescent quantum dots/ethidium bromide nanohybrids based on photoinduced electron transfer for DNA detection. Spectrochim. Acta Part A Mol Biomol Spectrosc.

[13] Raichlin S, Sharon E, Freeman R, Tzfati Y, Willner I (2011). Electron-transfer quenching of nucleic acid-functionalized CdSe/ZnS quantum dots by doxorubicin: a versatile system for the optical detection of DNA, aptamer-substrate complexes and telomerase activity. Biosens Bioelectron.

[14] Bisht S, Maitra A (2009). Dextran–doxorubicin/chitosan nanoparticles for solid tumor therapy. Nanomedicine Nanobiotechnology.

[15] Mei LT, Choong PFM, Dass CR (2009). Review: doxorubicin delivery systems based on chitosan for cancer therapy. J Pharm Pharmacol.

[16] Peng F, Su Y, Ji X, Zhong Y (2014). Doxorubicin-loaded silicon nanowires for the treatment of drug-resistant cancer cells. Biomaterials.

[17] Vaccari L, Canton D, Zaffaroni N, Villa R (2006). Porous Silicon as Drug Carrier for Controlled Delivery of Doxorubicin Anticancer Agent. Microelectron Eng.

[18] Liu MY, Zeng GJ, Wang K, Wan Q, Tao L, Zhang XY, Wei Y (2016). Recent developments in polydopamine: an emerging soft matter for surface modification and biomedic-al applications. Nanoscale.

[19] Liu MY, Ji JZ, Zhang XY, Zhang XQ, Yang B, Deng FJ, Li Z, Wang K, Yang Y (2015) Self-polymerization of dopamine and polyethyleneimine: novel fluorescent organic nanoprobes for biological imaging applications. 3:3476–3482 10.1039/c4tb02067g 32262230

[20] Shi YG, Liu MY, Deng FJ, Zeng GJ, Wan Q, Zhang XY (2017). Recent progress and development on polymeric nanomaterials for photothermal therapy: a brief overview. J Mater Chem B.

[21] Zhang XY, Zhang XQ, Yang B, Liu MY, Liu WY, Chen YW, Wei Y (2014). Polymerizable aggregation-induced emission dye-based fluorescent nanoparticles for cell imaging applications. Polym Chem.

[22] Zhang XY, Zhang XQ, Yang B, Liu MY, Liu WY, Chen YW, Wei Y (2014). Fabrication of aggregation induced emission dye-based fluorescent organic nanoparticles via emulsion polymerization and their cell imaging applications. Polym Chem.

[23] Long Z, Mao LC, Liu MY, Wan Q, Wan YQ, Zhang XY, Wei Y (2017). Marrying multicomponent reactions and aggregation-induced emission (AIE): new directions for fluorescent nanoprobes. Polym Chem.

[24] Zhang XY, Wang K, Liu MY, Zhang XQ, Tao L, Chen YW, Wei Y (2015). Polymeric AIE-based nanoprobes for biomedical applications: recent advances and perspectives. Nanoscale.

[25] Aryal S, Grailer JJ, Pilla S, Steeber DA, Gong S (2009). Doxorubicin conjugated gold nanoparticles as water-soluble and pH-responsive anticancer drug nanocarriers. J Mater Chem.

[26] Kumar SA, Peter YA, Nadeau JL (2008). Facile biosynthesis, separation and conjugation of gold nanoparticles to doxorubicin. Nanotechnology.

[27] Kang B, Afifi MM, Austin LA (2013). Elsayed, exploiting the nanoparticle plasmon effect: observing drug delivery dynamics in single cells via Raman/fluorescence imaging spectroscopy. ACS Nano.

[28] Chen H, Li B, Ren X, Li S (2012). Multifunctional near-infrared-emitting nano-conjugates based on gold clusters for tumor imaging and therapy. Biomaterials.

[29] Dhamecha D, Jalalpure S (2015). Doxorubicin-functionalized gold nanoparticles: characterization and activity against human cancer cell lines. Process Biochem.

[30] Curry D, Cameron A, Macdonald B, Nganou C, Scheller H (2015). Adsorption of doxorubicin on citrate-capped gold nanoparticles: Insights into Engineering Potent Chemotherapeutic Delivery Systems. Nanoscale.

[31] Kang B, Austin LA, El-Sayed MA (2012). Real-time molecular imaging throughout the entire cell cycle by targeted plasmonic-enhanced Rayleigh/raman spectroscopy. Nano Lett.

[32] Kang B, Mackey MA, Elsayed MA (2010). Nuclear targeting of gold nanoparticles in cancer cells induces dna damage, causing cytokinesis arrest and apoptosis. J Am Chem Soc.

[33] Yin HQ, Mai DS, Gan F, Chen XJ (2014). One-step synthesis of linear and cyclic RGD conjugated gold nanoparticles for tumour targeting and imaging. Rsc Adv.

[34] Bi F, Yin H, Zheng S, Gan F, Chen X (2016). One-step synthesis of peptide conjugated gold nanoclusters for the high expression of FGFR2 tumor targeting and imaging. Rsc Adv.

[35] Yin HQ, Bi FL, Gan F (2015). Rapid synthesis of cyclic RGD conjugated gold nanoclusters for targeting and fluorescence imaging of melanoma A375 Cells. Bioconjug Chem.

[36] Wang Y, Cui Y, Zhao Y, Liu R, Sun Z, Li W, Gao X (2012). Bifunctional peptides that precisely biomineralize Au clusters and specifically stain cell nuclei. Chem Commun.

[37] Xie J, Zheng Y, Ying JY (2009). Protein-directed synthesis of highly fluorescent gold nanoclusters. J Am Chem Soc.

[38] Tan YN, Lee JY, Wang DI (2010). Uncovering the design rules for peptide synthesis of metal nanoparticles. J Am Chem Soc.

[39] Yang F, Teves SS, Kemp CJ, Henikoff S (2014). Doxorubicin, DNA torsion, and chromatin dynamics. Biochim Biophys Acta.

[40] Dulkeith E, Morteani AC, Niedereichholz T, Klar TA, Levi SA (2002). Fluorescence quenching of dye molecules near gold nanoparticles: radiative and nonradiative effects. Phys Rev Lett.

[41] Schneider G, Decher G, Nerambourg N, Praho R, Werts MH (2006). Distance-dependent fluorescence quenching on gold nanoparticles ensheathed with layer-by-layer assembled polyelectrolytes. Nano Lett.

[42] Lu R, Gan W, Wu BH, Zhang Z, Guo Y, Wang HF (2005). C-H Stretching vibrations of methyl, methylene and methine groups at the vapor/alcohol (N = 1-8) interfaces. J Phys Chem B.

[43] Treybig DS, Potter JL (1988) Novel compositions prepared from alkyl substituted nitrogen-containing aromatic heterocyclic compounds and dicarboxylic acid monoanhydrides, US

[44] Zhu KJ, Kwei TK, Pearce EM (1989). Thermostability of Poly(p-Hydroxystyrene) blends with Poly(vinyl Pyrrolidone) and Poly(ethyl Oxazoline). J Appl Polym Sci.

[45] Margoshes M, Fassel VA (1955). The infrared spectra of aromatic compounds : I. the out-of-plane C-H bending vibrations in the region 625-900 cm^-1^. Spectrochim Acta.

[46] Lin X, Zhou M, Wang S, Lou H, Yang D, Qiu X (2014). Synthesis, structure, and dispersion property of a novel lignin-based polyoxyethylene ether from kraft lignin and poly(ethylene Glycol). Acs Sustain Chem Eng.

[47] Ayre A, Lalitha KG, Ruckmani K, Khutle N, Pawar H, Dand N, Vijaya C (2014). ICH Q8 Guidelines in practice: spray drying process optimization by 23 factorial design for the production of famotidine nanoparticles. Pharm Nanotechnol.

[48] Weng S, Wu J, Xu G (1989). FT-IR spectra of The C=O and C-H stretching vibration of lauric acid. Intl Conf on Fourier and Computerized Infrared Spectroscopy.

[49] Liu H, Shen M, Zhao J, Guo R, Cao X, Zhang G, Shi X (2012). Tunable synthesis and acetylation of dendrimer-entrapped or dendrimer-stabilized gold-silver alloy nanoparticles. Colloids Surfaces B Biointerfaces.

[50] Diao Y, Han W, Zhao H, Zhu S, Liu X, Feng X, Gu J, Yao C, Liu S, Sun C (2012). Designed synthetic analogs of the α-helical peptide temporin-La with improved antitumor efficacies via charge modification and incorporation of the integrin α_v_β_3_ Homing domain. J Pept Sci.

[51] Kim YH, Jeon J, Hong SH, Rhim WK, Lee YS (2011). Tumor targeting and imaging using cyclic RGD-PEGylated gold nanoparticle probes with directly conjugated iodine-125. Small.

[52] Fang J, Qin H (2012). Carbon monoxide, generated by heme oxygenase-1, mediates the enhanced permeability and retention effect in solid tumors. Cancer Sci.

