# Supplementary material for: One-step, Rapid and Green Synthesis of Multifunctional Gold Nanoparticles for Tumor-Targeted Imaging and Therapy
Source: Nanoscale Res Lett. 2020 Jan 31;15:29. doi: 10.1186/s11671-019-3232-3 (PMC6994604; doi:10.1186/s11671-019-3232-3)
Supplement: Supplementary file 1 — Additional file 1: Figure S1. TEM images of the NLS-GNPs, DRN-GNPs, DR-GNPs and DN-GNPs. Figure S2. UV-Visible absorption spectra of basic DOX, RGD peptides, NLS peptides and the supernatant of DN-GNPs and DR-GNPs. Figure S3. XPS spectra of the S from NLS–GNPs, DN-GNPs and DRN-GNPs. Figure S4. XPS spectra of the C, N, O from DOX-GNPs, NLS–GNPs, DR-GNPs, DN-GNPs, and DRN-GNPs. Figure S5. XRD figures of DOX-GNPs, NLS-GNPs, DR-GNPs, DN-GNPs and DRN-GNPs. Figure S6. The dynamic light scattering and zeta potential figures of the GNPs. Figure S7. TEM images of DRN-GNPs incubated in Hela and MCF-7 cells for 24 h. [file 11671_2019_3232_MOESM1_ESM.docx]

Supporting Information

**One-step, rapid and green synthesis of multifunctional gold nanoparticles for tumor targeted imaging and therapy**

*Hua Qin Yin,^a^ Guang Shao,^a^ Feng Gan^*a^ and and Gang Ye^*b^*

^a^ School of Chemistry, Sun Yat-Sen University, Guangzhou 510275, P.R. China. E-mail: cesgf@mail.sysu.edu.cn

^b^ Department of Gastroenterology, the First Affiliated Hospital of Jinan University, Guangzhou 510630, P.R. China

**Table of Contents:**

Figure S1. TEM images of the NLS-GNPs, DRN-GNPs, DR-GNPs and DN-GNPs．

Figure S2. UV-Visible absorption spectra of basic DOX, RGD peptides, NLS peptides and the supernatant of DN-GNPs and DR-GNPs.

Figure S3. XPS spectra of the S from NLS–GNPs, DN-GNPs and DRN-GNPs.

Figure S4. XPS spectra of the C, N, O from DOX-GNPs, NLS–GNPs, DR-GNPs, DN-GNPs, and DRN-GNPs.

Figure S5. XRD figures of DOX-GNPs, NLS-GNPs, DR-GNPs, DN-GNPs and DRN-GNPs.

Figure S6. The dynamic light scattering and zeta potential figures of the GNPs.

Figure S7. TEM images of DRN-GNPs incubated in Hela and MCF-7 cells for 24 h.


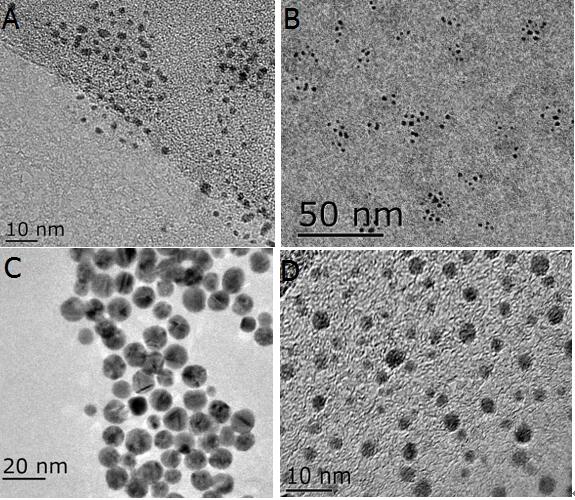


Figure S1. TEM images of the NLS-GNPs(A), DRN-GNPs(B), DR-GNPs (C)and DN-GNPs(D)．


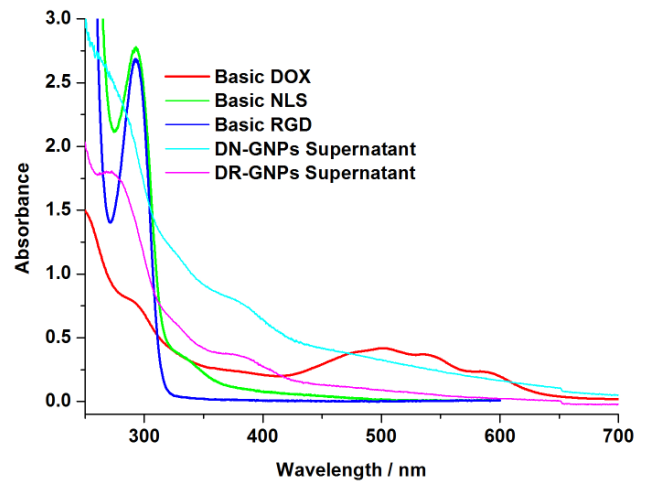


Figure S2. UV-Visible absorption spectra of basic DOX, RGD peptides, NLS peptides and the supernatant of DN-GNPs and DR-GNPs.


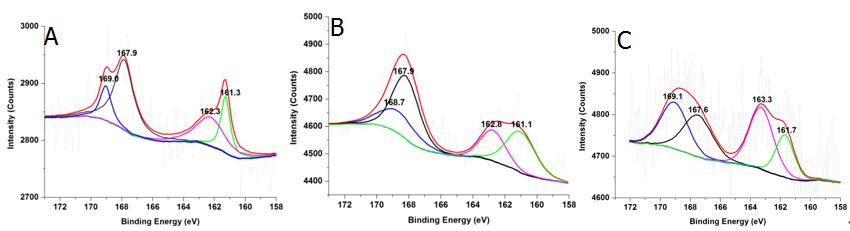


Figure S3. XPS spectra of the S from NLS–GNPs, DN-GNPs and DRN-GNPs.


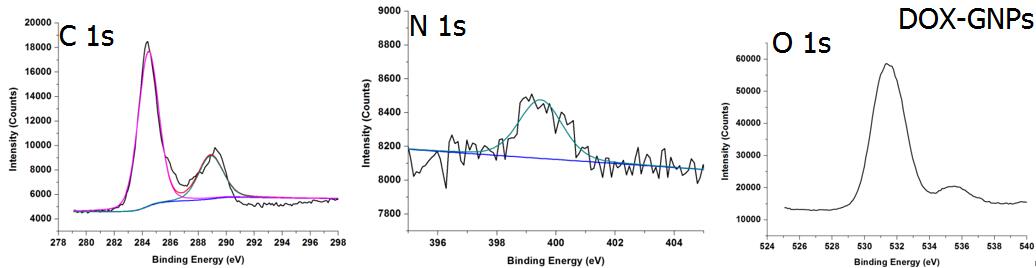


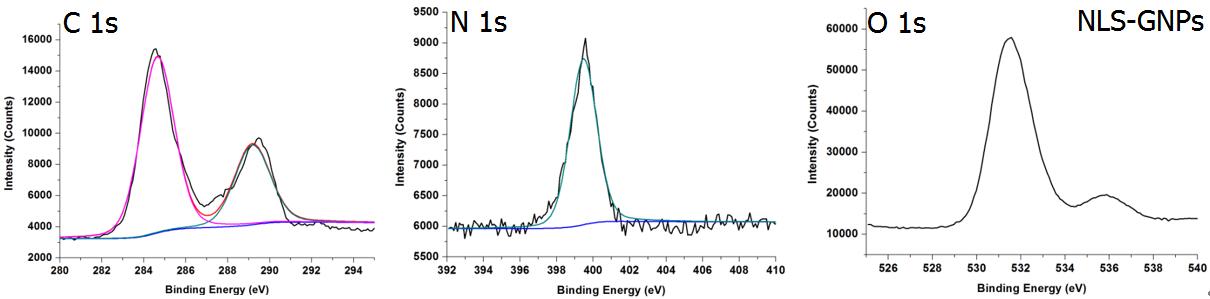


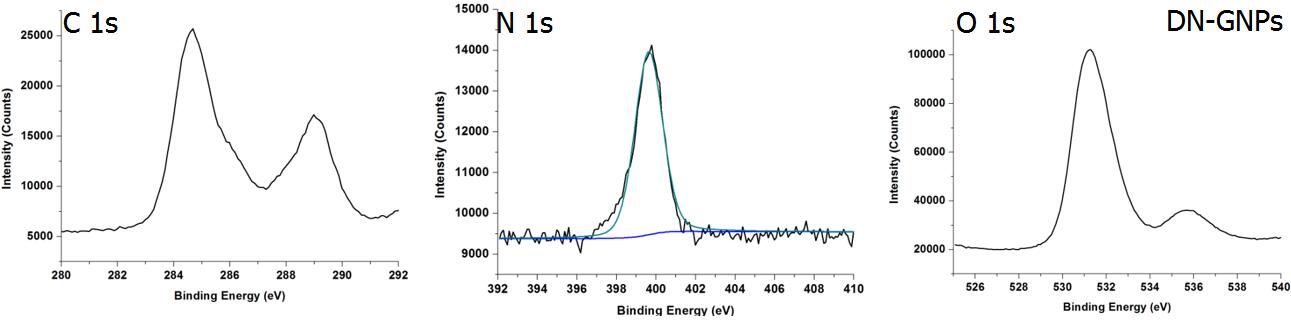


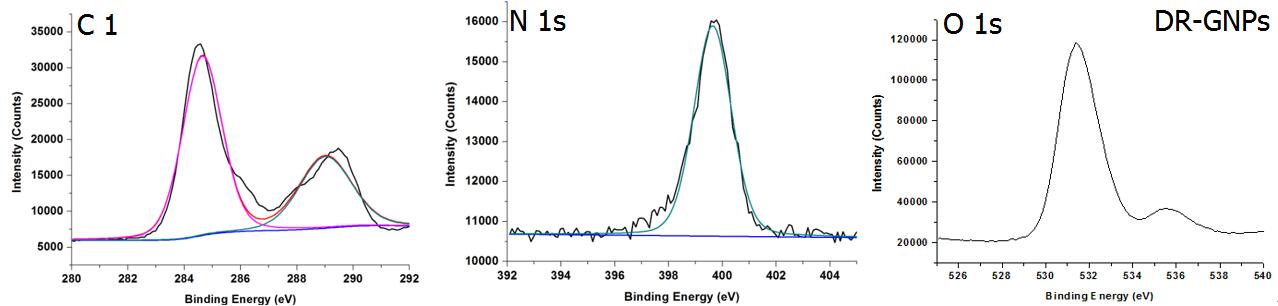


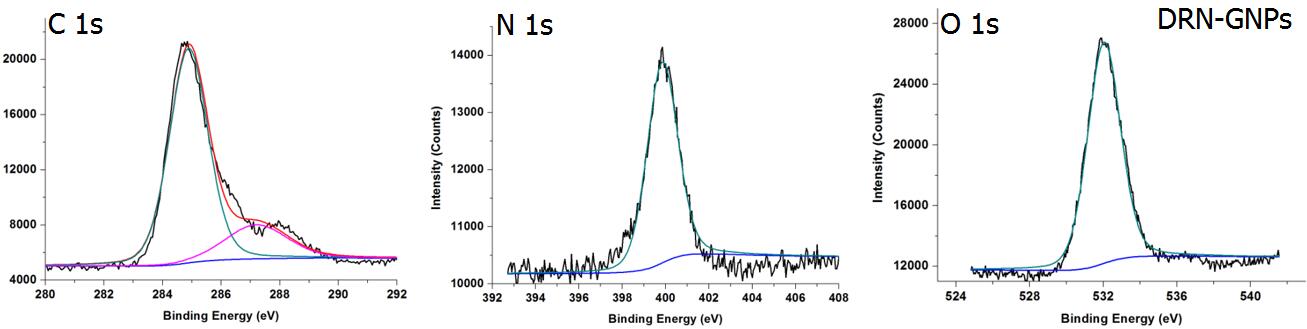


Figure S4. XPS spectra of the C, N, O from DOX-GNPs, NLS–GNPs, DN-GNPs, DR-GNPs and DRN-GNPs.


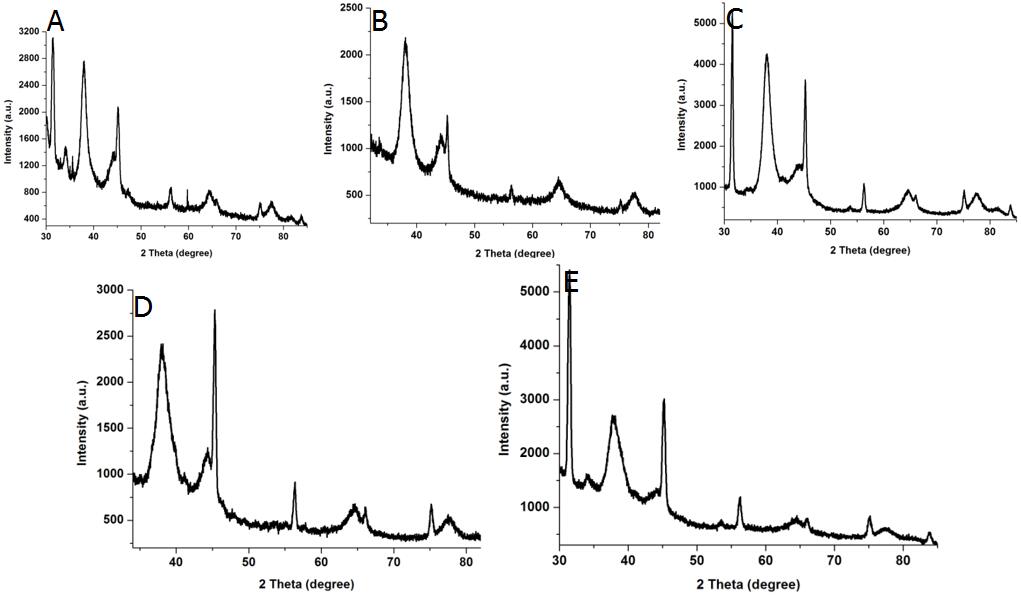


Figure S5. XRD figures of DOX-GNPs (A), NLS-GNPs (B), DR-GNPs (C), DN-GNPs (D) and DRN-GNPs (E).


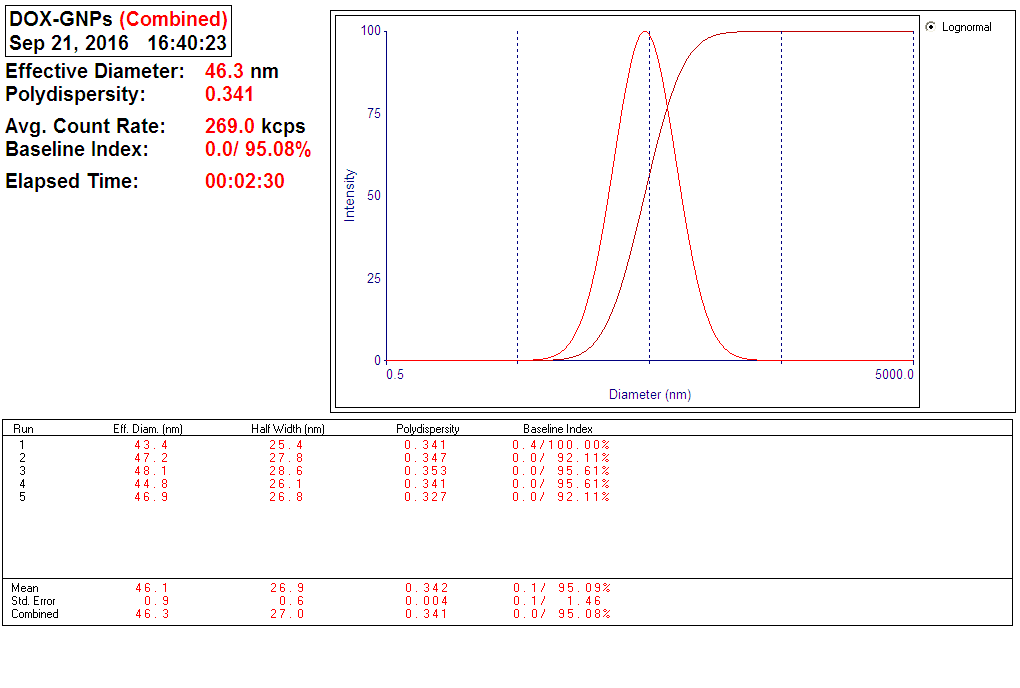

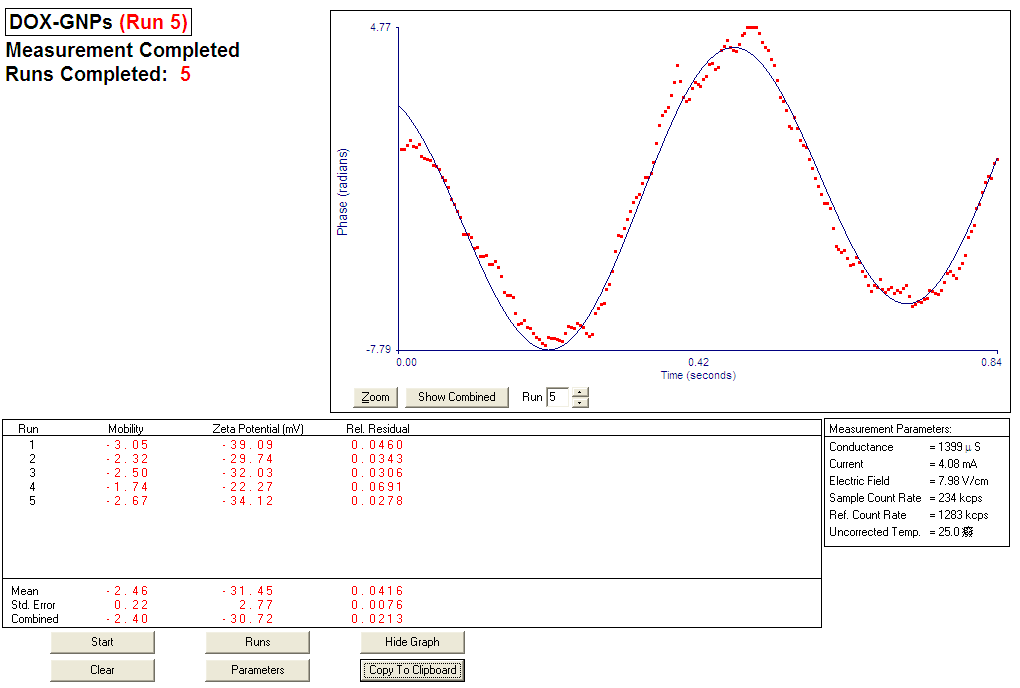


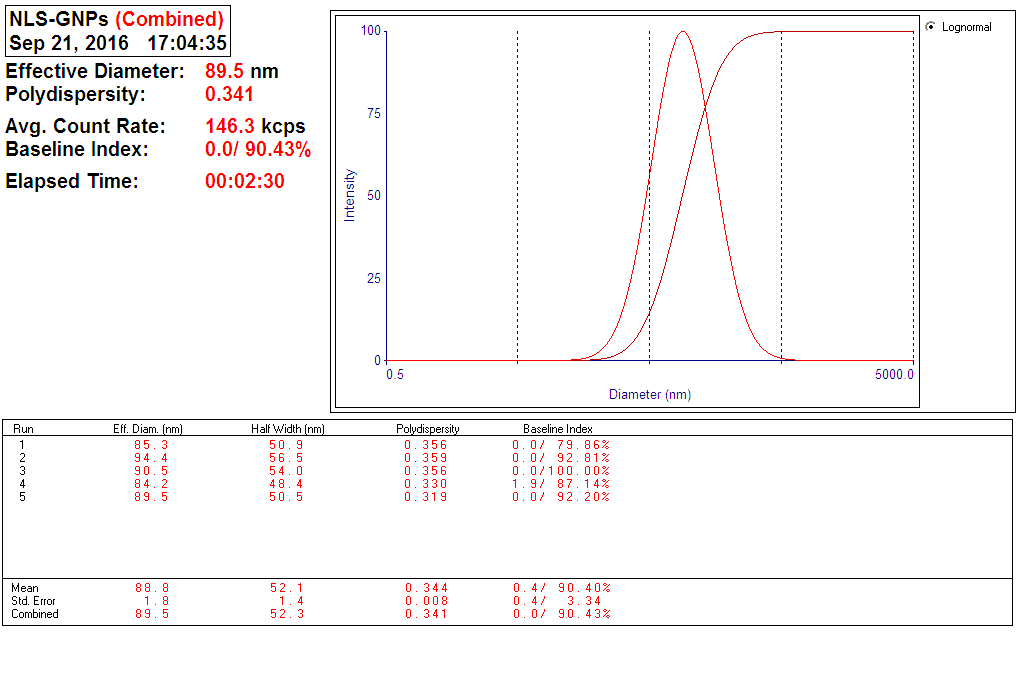

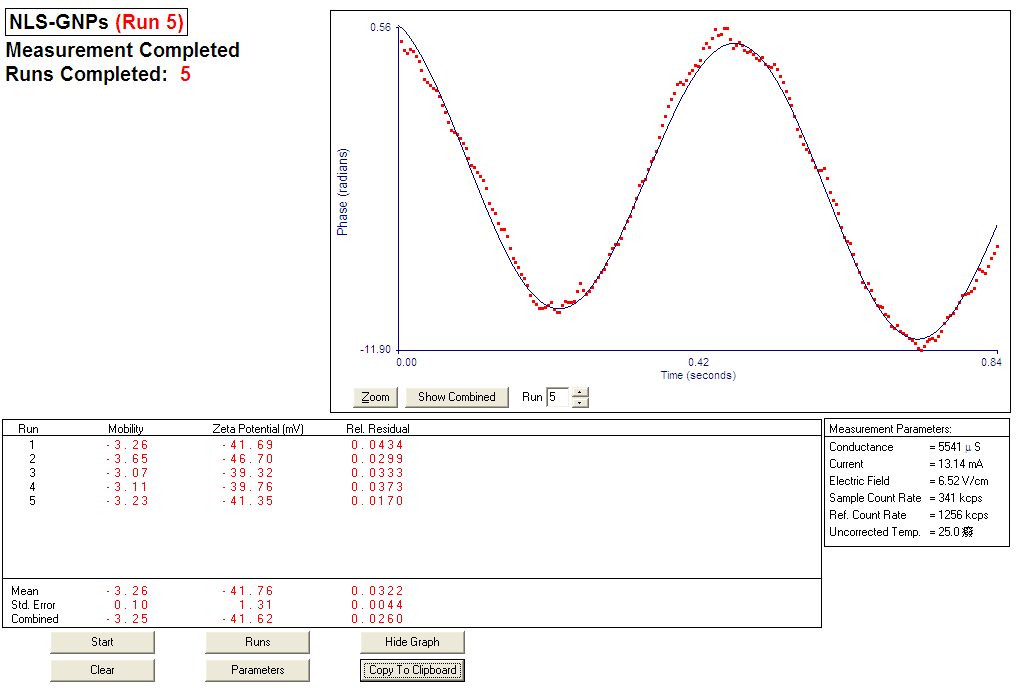


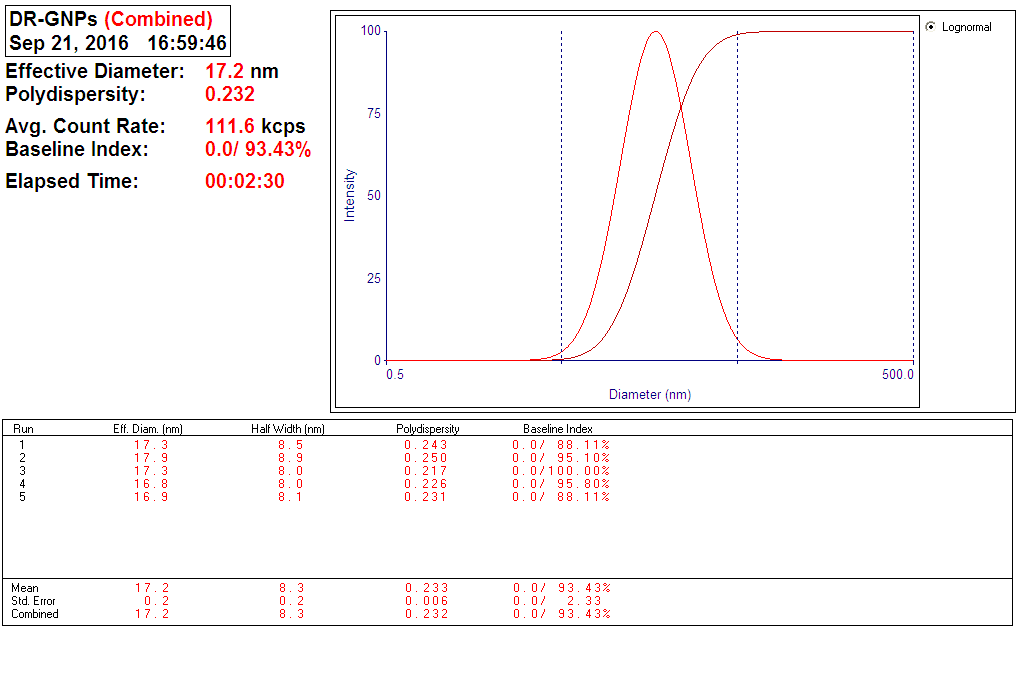

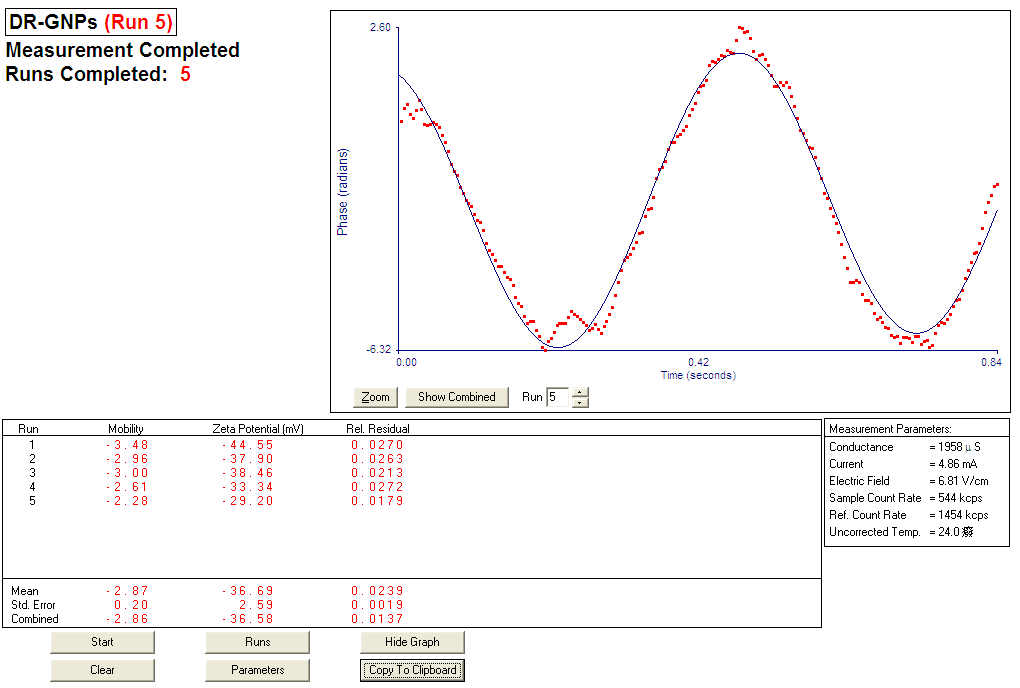


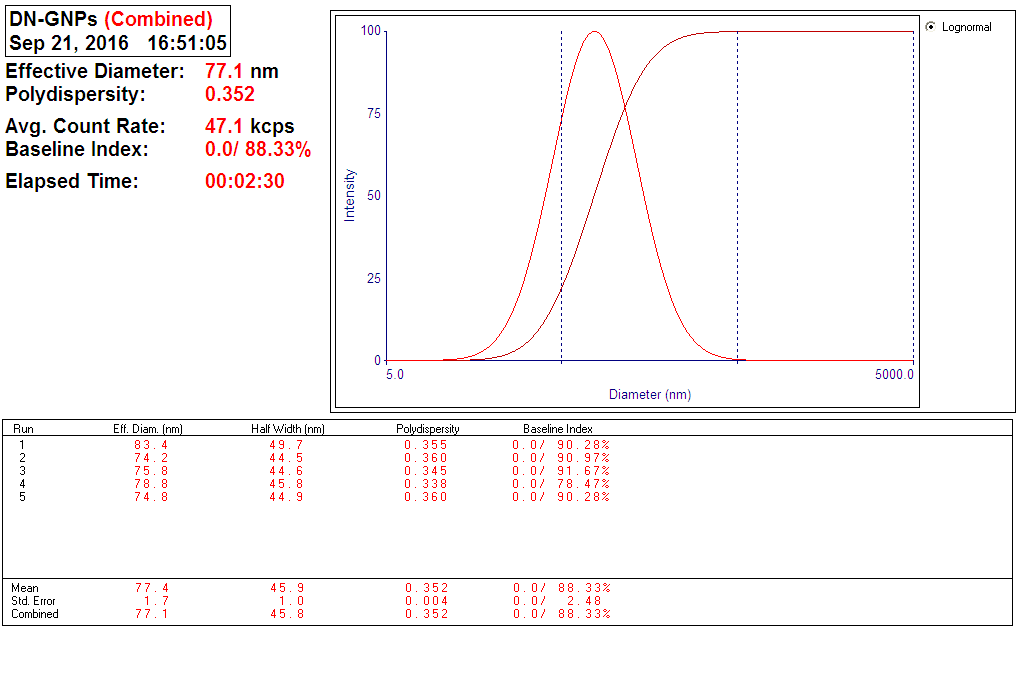

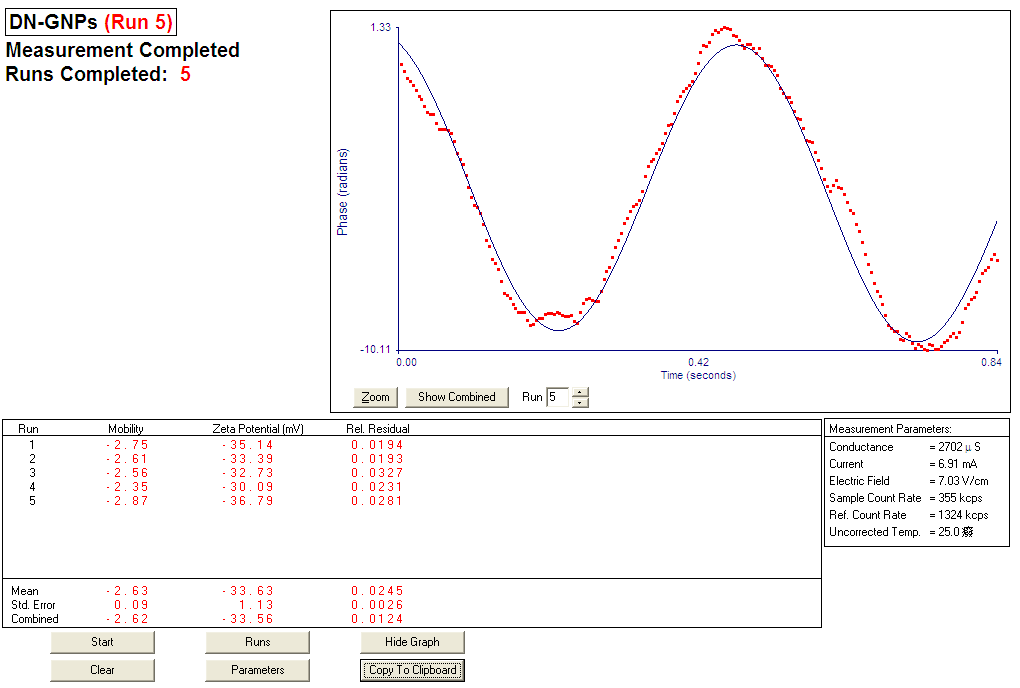


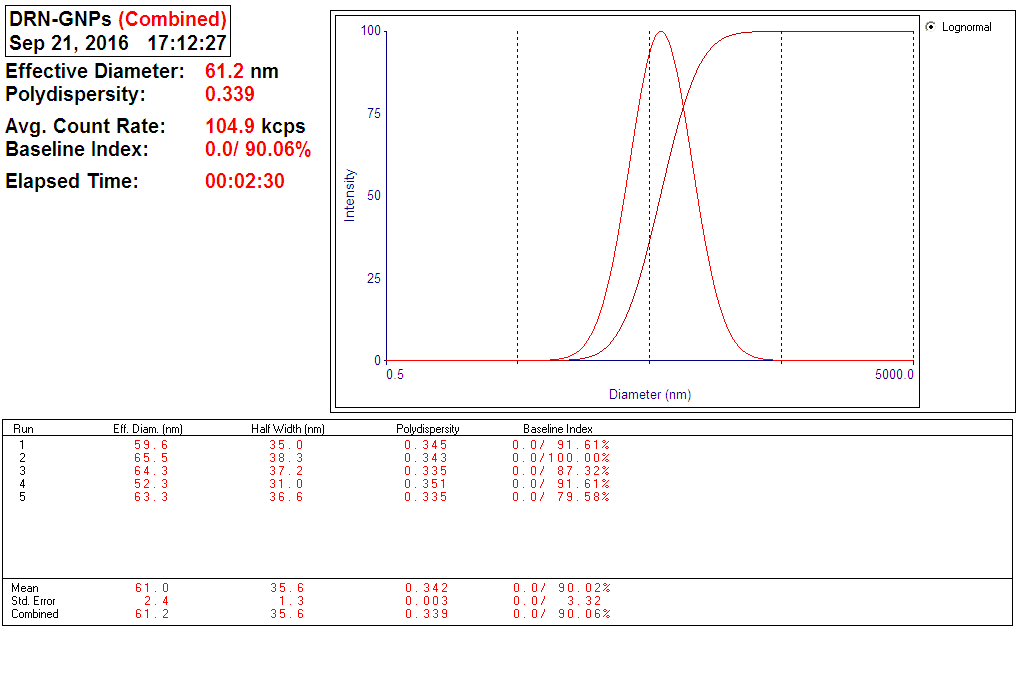

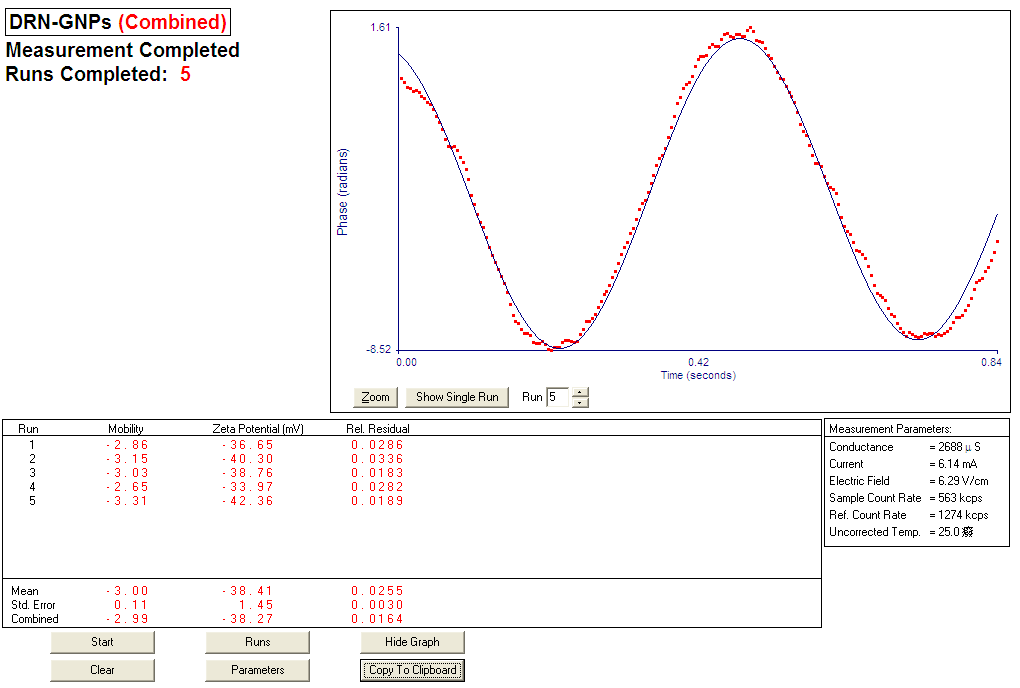


Figure S6. The dynamic light scattering and zeta potential figures of the GNPs.


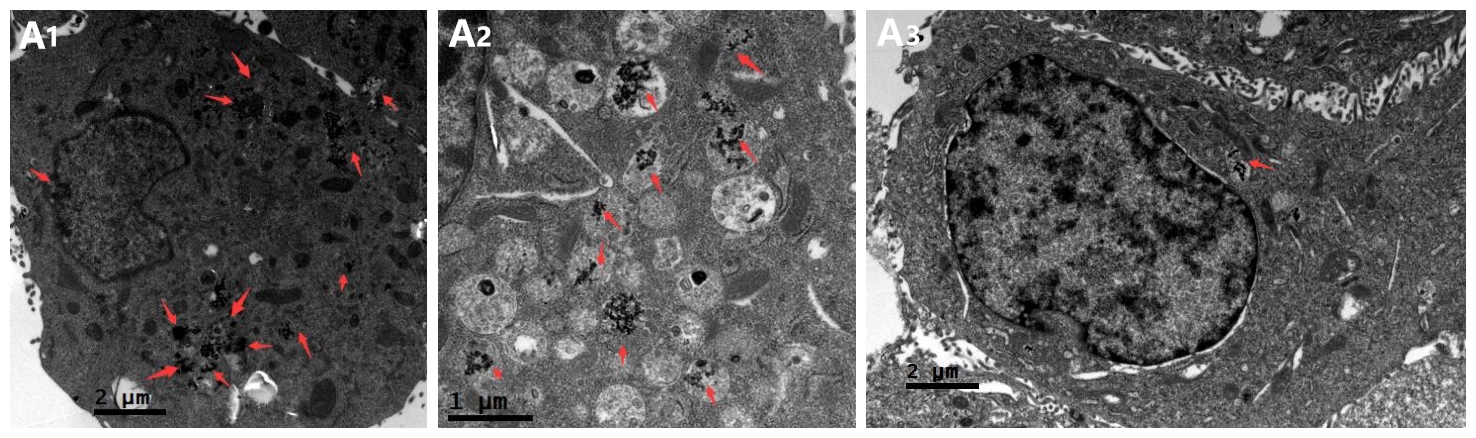


Figure S7. TEM images of DRN-GNPs incubated in Hela（A_1_, A_2_）and MCF-7 cells（A_3_）for 24 h.
